# Comparison of ventral organ development across Pycnogonida (Arthropoda, Chelicerata) provides evidence for a plesiomorphic mode of late neurogenesis in sea spiders and myriapods

**DOI:** 10.1186/s12862-018-1150-0

**Published:** 2018-04-05

**Authors:** Georg Brenneis, Gerhard Scholtz, Barbara S. Beltz

**Affiliations:** 1 Wellesley College, Neuroscience Program, 106 Central Street, Wellesley, MA 02481 USA; 2 Humboldt-Universität zu Berlin, Institut für Biologie, Vergleichende Zoologie, Philippstraße 13, Haus 2, 10115 Berlin, Germany

**Keywords:** Nervous system, Ventral nerve cord, Cell proliferation, Evolution, 5-bromo-2′-deoxyuridine, 5-ethynyl-2′-deoxyuridine, Callipallenidae, Ammotheidae, Pycnogonidae, Phoxichilidiidae

## Abstract

**Background:**

Comparative studies of neuroanatomy and neurodevelopment provide valuable information for phylogenetic inference. Beyond that, they reveal transformations of neuroanatomical structures during animal evolution and modifications in the developmental processes that have shaped these structures. In the extremely diverse Arthropoda, such comparative studies contribute with ever-increasing structural resolution and taxon coverage to our understanding of nervous system evolution. However, at the neurodevelopmental level, in-depth data remain still largely confined to comparably few laboratory model organisms. Therefore, we studied postembryonic neurogenesis in six species of the bizarre Pycnogonida (sea spiders), which – as the likely sister group of all remaining chelicerates – promise to illuminate neurodevelopmental changes in the chelicerate lineage.

**Results:**

We performed in vivo cell proliferation experiments with the thymidine analogs 5-bromo-2′-deoxyuridine and 5-ethynl-2′-deoxyuridine coupled to fluorescent histochemical staining and immunolabeling, in order to compare ventral nerve cord anatomy and to localize and characterize centers of postembryonic neurogenesis. We report interspecific differences in the architecture of the subesophageal ganglion (SEG) and show the presence of segmental “ventral organs” (VOs) that act as centers of neural cell production during gangliogenesis. These VOs are either incorporated into the ganglionic soma cortex or found on the external ganglion surface. Despite this difference, several shared features support homology of the two VO types, including (1) a specific arrangement of the cells around a small central cavity, (2) the presence of asymmetrically dividing neural stem cell-like precursors, (3) the migration of newborn cells along corresponding pathways into the cortex, and (4) the same VO origin and formation earlier in development.

**Conclusions:**

Evaluation of our findings relative to current hypotheses on pycnogonid phylogeny resolves a bipartite SEG and internal VOs as plesiomorphic conditions in pycnogonids. Although chelicerate taxa other than Pycnogonida lack comparable VOs, they are a characteristic feature of myriapod gangliogenesis. Accordingly, we propose internal VOs with neurogenic function to be part of the ground pattern of Arthropoda. Further, our findings illustrate the importance of dense sampling in old arthropod lineages – even if as gross-anatomically uniform as Pycnogonida – in order to reliably differentiate plesiomorphic from apomorphic neurodevelopmental characteristics prior to outgroup comparison.

**Electronic supplementary material:**

The online version of this article (10.1186/s12862-018-1150-0) contains supplementary material, which is available to authorized users.

## Background

The astounding species diversity of arthropods and the many different forms of their segmented bodies have fascinated zoologists for many centuries. Resolving the phylogenetic relationships within this taxon and understanding the evolutionary transformations leading to its extreme morphological diversification has proved to be an enormous challenge, with some issues persisting to this day [1, 2]. With the advent of molecular phylogenetics and its rapid advances in the last two decades, some traditional morphology-based arthropod relationships have been seriously questioned. As a result of discrepancies between morphological and molecular analyses, (re)investigation of morphological character complexes has been intensified in order to critically evaluate the evidence for debated nodes in the arthropod tree. For instance, scrutiny of different features pertaining to nervous system development and adult neuroanatomy (e.g., [3–6]) helped to clarify the relationships of the major mandibulate groups Myriapoda, Hexapoda and crustaceans: in agreement with molecular evidence (e.g., [7–9]), the neural characters consolidated support for the taxon Tetraconata (paraphyletic crustaceans + Hexapoda), thereby overhauling the traditionally advocated clade of myriapods and hexapods (see [1] for review). Over the last decades, the assessment and interpretation of neuroanatomical and neurodevelopmental character complexes in the context of arthropod phylogeny and evolution – more recently even in fossil representatives – has morphed into a prospering field, occasionally referred to as “neurophylogeny” or “neural cladistics” [10–17].

In this context, numerous studies on initial embryonic neurogenesis in chelicerate and mandibulate arthropods and their close relatives Onychophora have provided us with many details on the cell types and dynamics involved, as well as the gene network governing the neurogenic processes (see [18–20] for reviews). Yet, recent investigations of neurogenic processes in more advanced developmental stages – including free-living postembryonic instars in groups with anamorphic development – are still restricted to few taxa outside of Hexapoda. Especially the paucity of data in representatives of the chelicerate and myriapod lineages hampers well-founded comparison of late neurogenesis across arthropods.

**Fig. 1:**
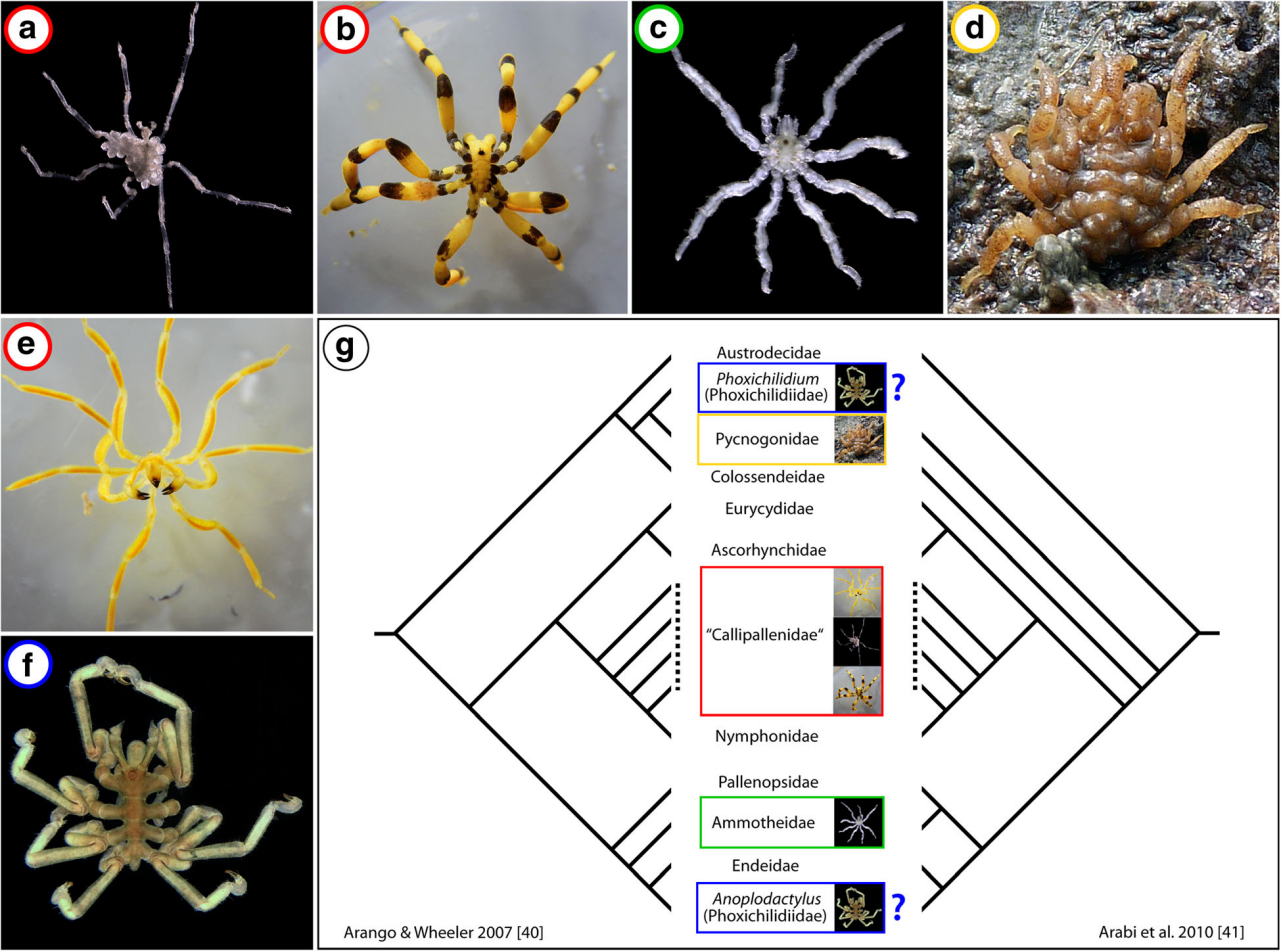
Investigated pycnogonid species and two hypotheses on their phylogenetic relationships. **a** *Callipallene brevirostris* (Johnston, 1837). Adult male bearing egg packages, dorsal view, 70% ethanol preservation. **b** *Stylopallene cheilorhynchus* Clark, 1963. Dorsal view of live female. **c** *Tanystylum orbiculare* Wilson, 1878. Adult female, dorsal view, 70% ethanol preservation. **d** *Pycnogonum litorale* Strøm, 1762. Male clinging to female during copula, dorsal view. **e** *Meridionale* sp. (member of “variabilis”-complex sensu Arango and Brenneis [45]). Antero-ventral view of live female. **f** *Phoxichilidium femoratum* (Rathke, 1799). Adult male, dorsal view, 70% ethanol preservation. **g** Distribution of the studied species shown against the backbone of two competing hypotheses of pycnogonid relationships [40, 41]. Note that the position of the genus *Phoxichilidium* remains questionable in the left cladogram, owing to its rather surprising separation from the genus *Anoplodactylus*. Both genera are generally accepted to constitute the morphologically and developmentally strongly supported taxon Phoxichilidiidae

As a first step to address this issue, our recent study on the pycnogonid *Meridionale* sp. (formerly known as *Pseudopallene* sp.; see [21]; Fig. 1e) followed the neurogenic processes in the ventral nerve cord (VNC) during the postembryonic developmental phase [22]. Pycnogonida (sea spiders) is an old lineage of marine arthropods dating back at least to the Ordovician [23], if not even to the Cambrian [24, 25]. Notably, it is currently considered the sister group to all other extant chelicerates [7, 26–28]. Owing to this phylogenetic position, Pycnogonida has been recognized as one of the crucial taxa to investigate at the morphological and developmental levels when seeking to reliably reconstruct plesiomorphic versus apomorphic features in the chelicerate lineage (e.g., [29]) and potentially even in arthropods in general. During the postembryonic development of *Meridionale* sp., the so-called “ventral organs” (VOs) are the locations of neurogenic cell proliferation in the VNC. In advanced postembryonic instars, these VOs represent bilaterally paired and segmentally arranged cell clusters that sit on the ventral surface of the ganglia and are connected to the ganglionic soma cortex via slender fibrous strands that penetrate through the neural sheath. Additional ganglion cells are produced in the ventral clusters and appear to move along the strands into the soma cortex – the strands accordingly acting as cell migration streams [22]. Importantly, however, despite Pycnogonida being a taxon with a comparably uniform gross architecture of the central nervous system (CNS) [30–32], classical developmental studies on representatives of different pycnogonid genera give deviating descriptions regarding the presence, exact position, and potential function(s) of the VOs [33–39]. These reported discrepancies necessitate further ingroup investigations beyond our recent single-species-approach, in order to confidently evaluate these neurodevelopmental characters of the taxon Pycnogonida in an arthropod-wide framework. Only such comparative studies guided by hypotheses on sea spider phylogeny enable reliable identification of plesiomorphic features of crown group Pycnogonida and meaningful comparison to the other arthropod lineages.

Hence, we set out to expand our understanding of the pycnogonid VOs and their function during postembryonic development in five pycnogonid species in addition to *Meridionale* sp., all of which belonging to different genera (Fig. 1). Two of these species are thought to be closely related to *Meridionale* sp. (Fig. 1a, b) with which they are traditionally placed in the Callipallenidae that may, however, represent a paraphyletic assemblage (Fig. 1g) (e.g., [40, 41]). The remaining three species are representatives of different taxa that are well-separated in the pycnogonid tree (Fig. 1c, d, f, g). In an attempt to maximize direct comparability of the semaphoronts examined, we focused on late postlarval and juvenile instars that are part of the postembryonic developmental pathways of all six species, in spite of distinct differences in earlier developmental periods (see [42] for review). Animals were exposed to in vivo cell proliferation markers followed by immunolabeling procedures to test whether or not (1) the presence and location of the VOs can be confirmed in the additional five species, (2) the characterization of postembryonic VOs as niches of neurogenic cell proliferation can be validated beyond the genus *Meridionale*, (3) any indications for specific neural precursor cell types can be found, and (4) cell migration patterns of newborn cells can be reconstructed. Results from these experiments are evaluated in the context of current hypotheses on sea spider phylogeny and plesiomorphic character states for crown group Pycnogonida are deduced and discussed within an arthropod-wide perspective.

## Methods

### Terminology

All species names of Pycnogonida have been updated according to the latest suggestions by Bamber and colleagues [43]. The designation of postembryonic stages as specific postlarval or juvenile instars follows the general definitions recently suggested by Brenneis and colleagues [42].

### Specimen collection

#### *Phoxichilidium femoratum* (Rathke, 1799), *Tanystylum orbiculare* Wilson, 1878, and *Callipallene brevirostris* (Johnston, 1837)

Specimens of *Phoxichilidium femoratum* (Phoxichilidiidae) were collected in June and July 2015 close to Prescott Park in Portsmouth, NH, USA. During the summer months, adults, juveniles and the last postlarval instar of this species cling to colonies of the hydrozoan *Tubularia larynx* [44]. Specimens of *Tanystylum orbiculare* (Ammotheidae) and *Callipallene brevirostris* (Callipallenidae) were collected in July 2015 and 2016 near Vineyard Haven and Menemsha on Martha’s Vineyard, MA, USA. At this time of year, these species are found in high abundances on colonies of the hydrozoan genus *Pennaria* as previously reported by Morgan [33]. Patches of the respective hydrozoan colonies were manually removed from floating docks and maintained for the duration of the experiments in the Animal Care Facility at Wellesley College. Small pieces of the colonies were screened under a stereomicroscope and pycnogonids together with some hydrozoans were manually transferred into 50 mL Falcon tubes with cut-out “windows” covered by a fine mesh to be left freely floating in a recirculating artificial sea water system.

#### *Stylopallene cheilorhynchus* Clark, 1963, and *Meridionale* sp.

Specimens of both callipallenid species were collected in November 2015 in coastal waters near Eaglehawk Neck, Tasmania, Australia. During SCUBA diving, animals were collected by hand from their bryozoan prey *Orthoscuticella* sp. For the duration of the experiments, pycnogonids were kept with patches of bryozoan colonies in small mesh cages submerged in aerated tanks filled with local natural sea water. All *Meridionale* specimens investigated are part of the “*variabilis*”-complex, the taxonomy of which being still unsatisfactorily resolved at the species level [45]. Only specimens of the morphotype with blackened chela and proboscis tips were used.

#### *Pycnogonum litorale* Strøm, 1762

Postlarval instars and juveniles of *P. litorale* (Pycnogonidae) were lab-reared in a recirculating artificial sea water system in Berlin, the setup of which being adapted from previous successful studies [46–49].

### In vivo cell proliferation experiments

Pycnogonids were transferred into 6-well plates filled with filtered natural sea water containing either the thymidine analog 5-bromo-2′-deoxyuridine (BrdU, 5 mg/mL; Sigma-Aldrich, #B5002) or 5-ethynyl-2′-deoxyuridine (EdU, 100 μM, provided in Click-iT® EdU Alexa Fluor® 488 or 594® Imaging Kits; Invitrogen – Molecular Probes® #C10337 or #C10339, respectively). The plates were slowly rotated on a horizontal shaker for the duration of the nucleoside exposure. Exposure times differed between experiments (see results for specific time periods). In double-nucleoside labeling experiments, BrdU was used as first marker, followed by EdU after a variable intervening time period in sea water tanks.

### Specimen fixation and pre-staining processing

After nucleoside exposure, animals were fixed for 1 to 2 nights at 4 °C in 4% PFA/SW (16% formaldehyde in double-distilled H_2_O (methanol-free, Electron Microscopy Sciences, #15710) diluted 1:4 in filtered natural sea water) and subsequently either directly processed or alternatively stored at − 20 °C in cryoprotectant buffer (0.05 M sodium phosphate buffer, 300 g/L sucrose, 30% (*v*/v) ethylene glycol, 10 g/L polyvinylpyrrolidone) until further use. In the majority of cases, the complete CNS was dissected in phosphate-buffered saline (PBS; 75 mM Na_2_HPO_4_, 20 mM KH_2_PO_4_, 100 mM NaCl, pH 7.4). In some specimens of *Meridionale* sp. and *P. litorale*, the walking legs, ovigers and the strongly sclerotized portions of the cheliphores (if present) were removed, the remaining animal briefly coated in 0.01% Poly-L-Lysine and embedded in 5% agar (dissolved in double-distilled H_2_O) for sectioning. Sagittal sections of 50 μm thickness were produced with a Leica VT 1000S vibratome.

Dissected CNSs and sections were thereafter exposed to 10 μg/mL Proteinase K (Ambion, #AM2546) in PBS for 10–15 min at 37 °C, followed by several quick rinses in PBS under gentle shaking at room temperature (RT). In the case of subsequent BrdU labeling, an additional incubation step in 2 N HCl for 20 min at RT was performed.

### Immunohistochemistry and fluorescent histochemistry

Prior to antibody exposure, samples were permeabilized in PBS + 0.3% Triton-X (PBTx) for at least 2 hours with several changes of the buffer. The primary and secondary antibodies were diluted in PBTx, with incubation times lasting 12–48 h at 4 °C. In developing and adult pycnogonid ganglia, immunolabeling of the structural protein acetylated α-tubulin can be used to visualize the neurites and part of the cortical cytoskeleton of the neuronal somata, thus giving an excellent overview of the ganglionic architecture (e.g., [22, 50]). A primary monoclonal mouse antibody (anti-ac-α-tub IgG 2b Isotype, clone 6-11 B-1, Sigma T6793, dilution 1:200) was used in conjunction with a Cy3- or Alexa Fluor®647-coupled secondary goat antibody (anti-mouse IgG (H + L), Jackson/Dianova, #115-165-146 (Cy3) & #115-605-166 (A647), dilution 1:200). For detection of BrdU-positive cells, a monoclonal rat anti-BrdU primary antibody was applied (Accurate Chemical & Scientific Corporation, clone BU1/75 (ICR1), # OBT0030G, dilution 1:50) followed by a Alexa Fluor®488-coupled goat anti-rat secondary antibody (IgG(H + L), Jackson/Dianova, #112-545-167, dilution 1:200). Detection of cells in M phase was performed with a polyclonal rabbit anti-phosphorylated histone H3 antibody (Upstate Biotechnology, Lake Placid, NY, Cat# 07-492, dilution 1:200) in combination with a Cy3-coupled goat anti-rabbit secondary antibody (IgG(H + L), Jackson/Dianova, #111-165-144, dilution 1:200). All antibody incubations were followed by rinsing in PBTx for at least 4 h at RT with gentle agitation on a horizontal shaker. As control for non-specific binding of the secondary antibodies, primary antibodies were omitted; this resulted in complete loss of signal.

EdU-positive cells were detected with Click-iT® EdU Alexa Fluor® 488 or 594® Imaging Kits (Invitrogen – Molecular Probes®, #C10337 or #C10339, respectively) according to the guidelines of the manufacturer’s protocol but with an extended Click-iT® reaction time of 2–3 h.

F-actin labeling for visualization of the cortical cytoskeleton in *Ph. femoratum* was performed with Alexa Fluor®488 phalloidin (Invitrogen – Molecular Probes® #A12379, 1:50 in PBTx) overnight at 4 °C.

Dissected CNSs of 70% ethanol-preserved adult specimens of the species *Nymphon gracile* Leach, 1814, *Parapallene australiensis* (Hoek, 1881), *Endeis spinosa* Montagu, 1808, and *Cilunculus japonicus* (Turpaeva, 1990) were labeled overnight at 4 °C with the lipophilic marker FM 1-43FX (Invitrogen Molecular Probes®, #F35355, 5 μg/mL in double-distilled H_2_O).

Nuclear counterstaining was performed with Hoechst (H33342, Invitrogen Molecular Probes®, #H1399, 1 μg/mL in PBS) after all other labeling procedures. Incubation lasted at least 1 h (sections) and was occasionally extended overnight at 4 °C (complete CNSs). After final rinsing in PBS, samples were cleared in Vectashield® Mounting Medium (Vector Laboratories, Inc.) and mounted on microscopic slides with tiny plasticine pieces attached to the corners of the cover slips to prevent compression of the samples.

### Data documentation, analysis and presentation

Images of adult specimens preserved in 70% ethanol are based on z-stacks that were manually taken with a Leica M165C FC stereomicroscope equipped with a Leica DFC310 FX camera. Images were thereafter aligned in Adobe Photoshop CS3. Fluorescent overview images of CNSs were taken with a Zeiss Lumar V12, aligned z-stacks being automatically created with Zeiss AxioVision software (Version 4.7.10). Each z-stack was merged to a single image with extended depth of field using Helicon Focus software (Heliconsoft, Version 6.7.1).

Confocal laser scanning microscopy of fluorescent labeling was performed with a Leica DMI 6000 CS microscope coupled to a Leica TCS SP5 II scan unit. Based on the excitation spectra of the applied fluorochromes, a combination of UV laser (405 nm → Hoechst), argon laser (488 nm → Alexa Fluor® 488) and helium-neon laser (543 nm → Cy™3; 594 nm → Alexa Fluor® 594; 633 nm → Alexa Fluor® 647) was selected for imaging.

The 3D reconstruction software Imaris (Bitplane AG, Version 7.0.0) was used for subsequent analyses. Software tools were applied as previously described (e.g., [19]). Global contrast and brightness values of some of the images were adjusted using Adobe Photoshop CS3. All figures were compiled with Adobe Illustrator CS3. Supplementary movies were generated in Imaris (Animation module) and subsequently transformed into MP4-format using the freeware FormatFactory (version 2.96, www.pcfreetime.com).

## Results

### Gross architecture of the VNC and its development in postlarval and juvenile instars

**Fig. 2:**
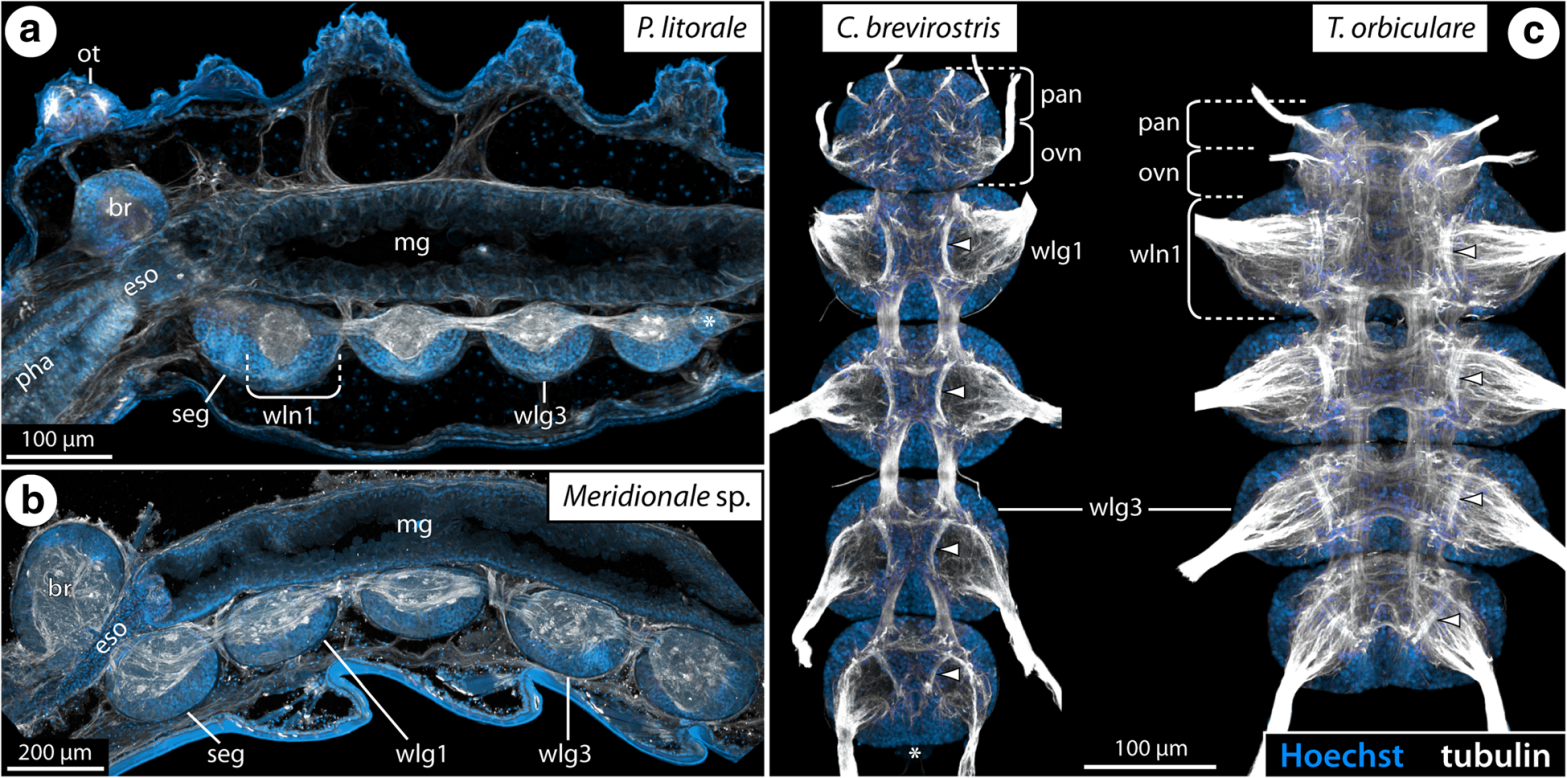
Gross anatomy of the adult VNC of four studied species. Immunolabeling of acetylated tubulin (white) with nuclear counterstain (blue), maximum projections of vibratome sections (**a**, **b**) or wholemount VNCs (**c**). Asterisks mark fused vestiges of the transient posterior ganglion anlagen. **a** *P. litorale*, sagittal section of first juvenile instar, anterior to the left. Note the inclusion of walking leg neuromere 1 in the subesophageal ganglion. **b** *Meridionale* sp., sagittal section of adult male, anterior to the left. Note the distinct anatomical separation of the subesophageal ganglion and the one of walking leg segment 1. **c** Adult VNC of *C. brevirostris* (left) and *T. orbiculare* (right), ventral view. Note anatomical separation of the subesophageal ganglion and walking leg ganglion 1 in *C. brevirostris*, but their fusion in the antero-posteriorly compressed VNC of *T. orbiculare*. Arrowheads highlight the ventral longitudinal tracts that are particularly conspicuous in the left VNC. Abbreviations: br – brain, eso – esophagus, mg – midgut, ot – ocular tubercle, ovn – ovigeral neuromere, pan – palpal neuromere, pha – pharynx, seg – subesophageal ganglion, wlg – walking leg ganglion, wln – walking leg neuromere

**Fig. 3:**
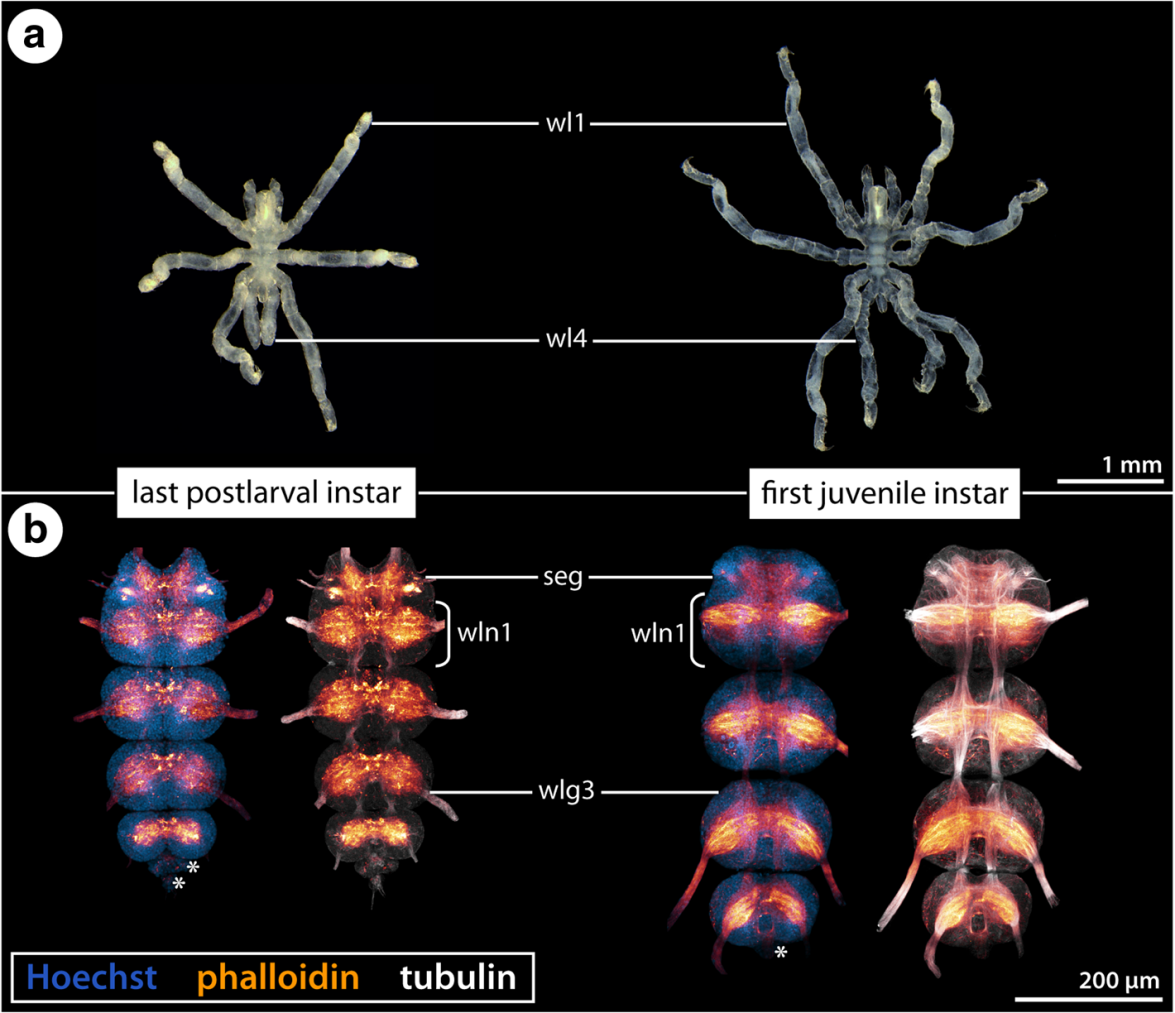
Last postlarval and first juvenile instars of *Ph. femoratum* and anatomy of their VNC. **a** Stereomicroscope images of the last postlarval instar (left) and the first juvenile instar (right), ventral view, anterior to the top, 70% ethanol preservation. Note incomplete differentiation of walking leg pair 4 in the last postlarval instar. **b** Maximum projections of wholemount VNCs of the last postlarval and first juvenile instars in ventral view, anterior to the top. Labeling of acetylated tubulin (white), phalloidin-F-actin (glow) and nuclear counterstain (blue). The decreasing size of the walking leg ganglia along an a-p developmental gradient is especially evident in the postlarval instar. Asterisks mark transient posterior ganglion anlagen. Abbreviations: seg – subesophageal ganglion, wl – walking leg, wlg – walking leg ganglion, wln – walking leg neuromere

The adult VNC of each of the six species investigated comprises a chain of ganglia that has been formed by the neuromeres of the palpal, the ovigeral and the four walking leg segments (Fig. 2). In the three callipallenid representatives (*Meridionale* sp., *S. cheilorhynchus*, *C. brevirostris*), the palpal and ovigeral neuromeres fuse during development into a bipartite subesophageal ganglion (SEG; Fig. 2b, c). In contrast to this, the SEG of the other three species (*Ph. femoratum*, *T. orbiculare*, *P. litorale*) is tripartite, encompassing not only of the palpal and ovigeral neuromeres but also the neuromere of walking leg segment 1 (Figs. 2a, c and 3b). The fusion of the three neuromeres begins in the early postlarval phase and is already well-advanced in the late postlarval and juvenile instars that are the focus of the present study. In both types of SEG, the neuronal somata are arranged in one contiguous cortex that surrounds the ganglionic neuropil. Within the neuropil delineation of the contributing segmental units remains possible, especially in the case of walking leg neuromere 1 in the tripartite SEG of *Ph. femoratum*, *T. orbiculare* and *P. litorale* (Figs. 2a, c and 3b).

In the last postlarval and the first juvenile instars, the developing ganglia of the walking leg segments are well-defined morphological units (e.g., Fig. 3b). However, as postembryonic development of all species – with the exception of *C. brevirostris* – is anamorphic, the size of the growing ganglia decreases along an anterior-posterior (a-p) developmental gradient, which is especially evident in the last postlarval instar (e.g., Fig. 3b). In this instar, the a-p gradient is also externally recognizable by the incompletely differentiated limb buds of walking leg pair 4 (Fig. 3a).

In addition to the walking leg ganglia, one or two small posterior ganglion anlagen are transiently formed during the postlarval phase but subsequently fuse with the last walking leg ganglion in juveniles (e.g., Figs. 2a and 3b; Additional file 1; see [22] for more details).

**Fig. 4:**
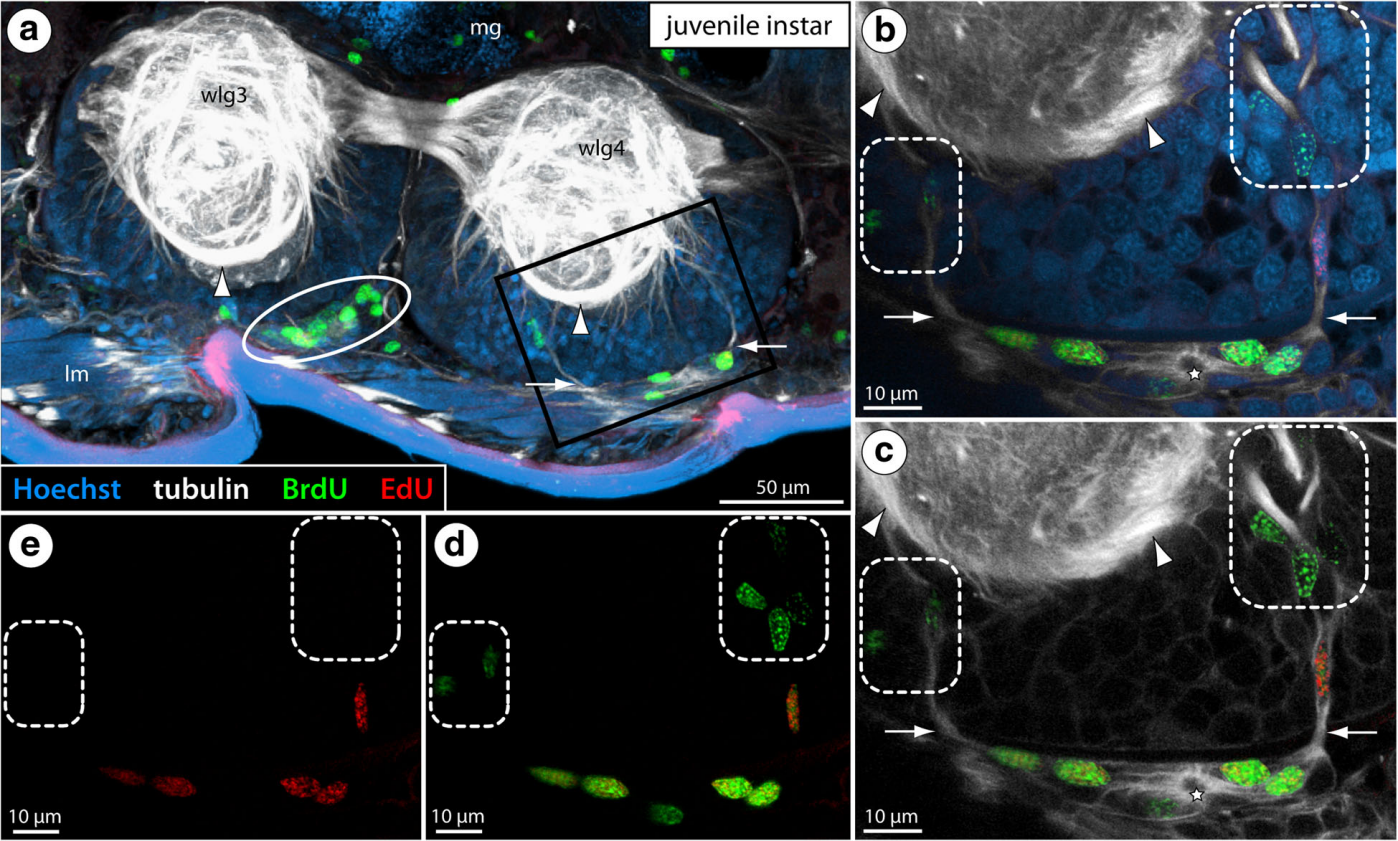
BrdU-EdU double-labeling in the VNC of a juvenile instar of *Meridionale* sp. Labeling of acetylated tubulin (white), BrdU (green) and EdU (red) with Hoechst nuclear counterstain (blue) after 5-day experiment (4 h BrdU exposure, 92 h sea water, 4 h EdU exposure, 20 h sea water, sacrifice). Sagittal vibratome section, anterior to the left. Arrows highlight the anterior and posterior migratory streams that penetrate through the neural sheath into the soma cortex. Arrowheads mark the ventral longitudinal tract. Stars indicate the small central cavity around which tubulin-rich cell processes converge. **a** Walking leg ganglia 3 and 4. The black rectangle indicates the region of interest shown in (**b**–**e**). The ellipse highlights the external VO-cluster of walking leg ganglion 3, featuring numerous BrdU-positive cells. **b**–**e** Detail of the external VO-cluster and the migratory streams penetrating into the cortex of walking leg ganglion 4. The images depict different marker combinations: **b** all four markers, **c** tubulin, BrdU and EdU, **d** BrdU and EdU, **e** EdU. Note the presence of exclusively BrdU-positive cells in deeper cortex layers (stippled areas). EdU-positive cells co-label for BrdU and are restricted to the external VO-cluster and the proximal part of the streams. Abbreviations: lm – longitudinal muscle bundles, mg – midgut, wlg – walking leg ganglion

Among the numerous longitudinal and transverse tracts in the walking leg ganglia of pycnogonids [50], one ventral longitudinal tract is particularly prominent in all studied species (e.g., Figs. 2c and 4a; Additional file 2). It encircles the ganglionic neuropil on its ventral surface and is positioned in line with the interganglionic connectives. This tract could be used as an important landmark for the cell proliferation studies.

### Confirmation of cell proliferation in the external VOs of *Meridionale* sp.

In *Meridionale* sp., only advanced juvenile instars were investigated (originally described as postembryonic stage 6, although this stage may encompass a number of subsequent instars [51]). Our previous study on this species described the development of the VOs and resolved their function via documentation of mitoses and cell counts in the ganglionic soma cortex [22]. Here, cell proliferation experiments were performed to independently validate previous results, study the direction of cell movement and enable direct comparison to the other pycnogonid species.

In corroboration of the earlier results, double-nucleoside labeling with BrdU and EdU (4 h BrdU exposure, 92 h sea water, 4 h EdU exposure, 20 h sea water, sacrifice = 5 d survival time) reveals the expected cell proliferation in the external VO-clusters and along the streams that penetrate through the neural sheath into the ganglionic soma cortex (Fig. 4; Additional file 2). Beyond that, the labeling profiles of nuclei within the VOs provide strong evidence that cell migration proceeds from the clusters into the soma cortex: Labeling for BrdU – the marker that was presented first – is found in the external VO-cluster, but in addition to this, almost a third of all BrdU-positive (BrdU+) cells are located in the streams and even deeper within the soma cortex (258 out of 857 BrdU+ cells [= 30.1%] in streams and cortices of six specimens) (Fig. 4b–d; Additional file 2). In contrast, the great majority of cells labeling for EdU – the marker presented second – remain confined to the external VO-clusters (352 out of 383 EdU+ cells [= 91.9%] in the clusters of six specimens) and only a low number of them is encountered in the proximal parts of the streams (Fig. 4e; Additional file 2). This pattern with its more restricted distribution of the EdU+ cells confirms the external VO-cluster as the predominant proliferation center, acting as a neurogenic niche for new neural precursor cells that subsequently migrate into the interior of the ganglion.

### Centers of cell proliferation in the postlarval and juvenile VNC of other pycnogonid species

In addition to juveniles of *Meridionale* sp., we conducted in vivo cell proliferation experiments with postlarval and/or juvenile instars of all other species except *P. litorale*.

**Fig. 5:**
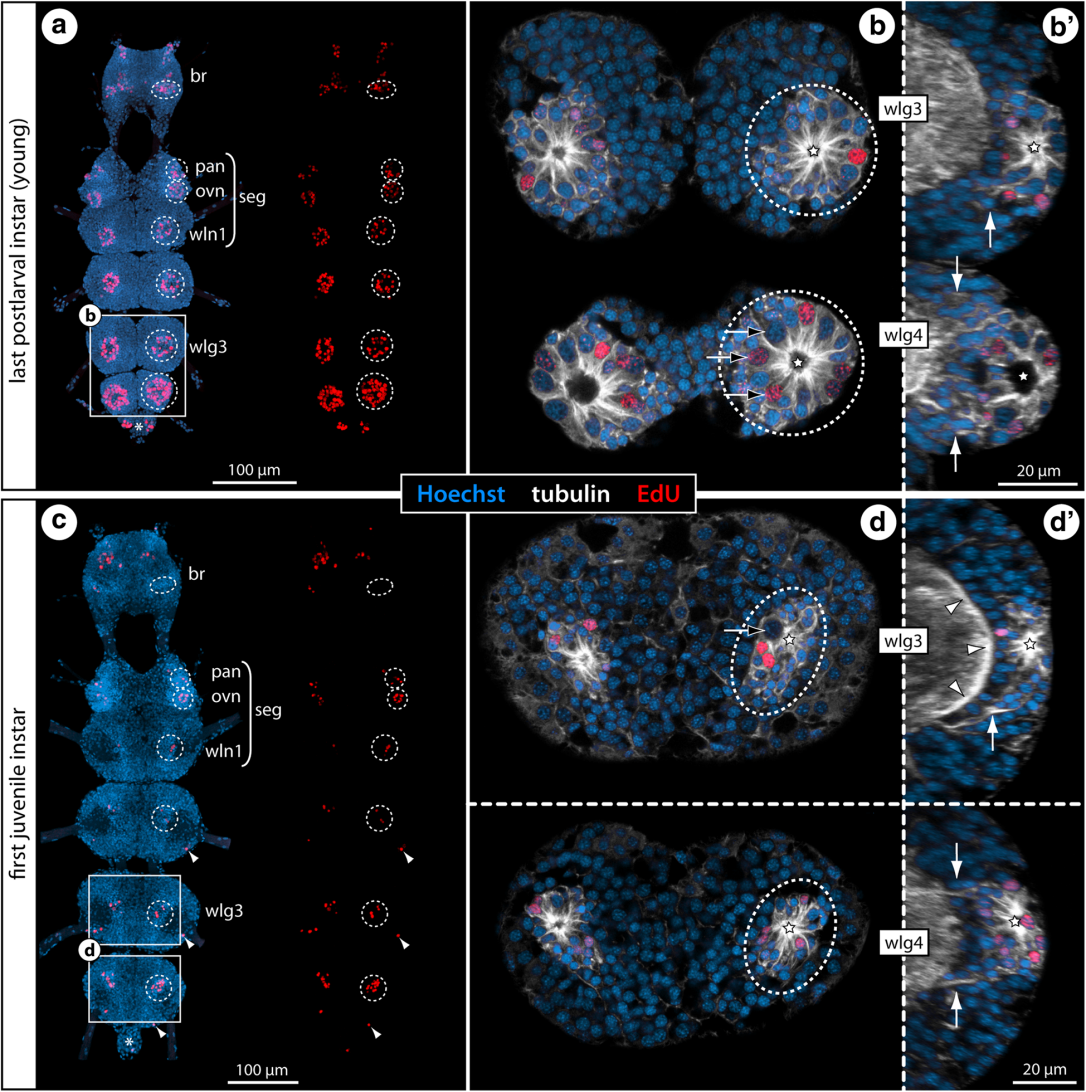
Cell proliferation in the VNC of postlarval and juvenile *Ph. femoratum* instars. Labeling of acetylated tubulin (white) and EdU (red) with nuclear counterstain (blue) after 4 h EdU exposure. Maximum projections (**a**, **c**) and optical sections (**b**, **b’**, **d**, **d’**) of wholemount CNSs, anterior to the top. Stippled ovals highlight the internal VOs of one body half. Stars indicate the central cavity around which tubulin-rich cell processes converge. White arrows indicate broad tubulin-rich cell bands (**b’**) and more condensed fibrous strands (**d’**) extending dorsally from the VOs towards the neuropil. Asterisks mark transient posterior ganglion anlagen. **a**: CNS of the last postlarval instar, ventral view, EdU labeling shown separately on the right. Note the gradual antero-posterior increase of cell proliferation. **b** & **b’**: Wlg3–4, horizontal and sagittal sections. Black arrows mark larger nuclei in the VOs of wlg4. Note also wider cavities of the VOs in wlg4. **c**: CNS of the first juvenile instar, ventral view, EdU labeling shown separately on the right. Arrowheads mark single EdU-positive cells in the periphery of the ganglia. **d** & **d’**: Wlg3–4, horizontal and sagittal sections. Only few large VO nuclei remain (black arrow), VO nuclei being generally smaller than the neuronal nuclei in the soma cortex. Arrowheads mark the ventral longitudinal tract in wlg3. Abbreviations: br – brain, ovn – ovigeral neuromere, pan – palpal neuromere, seg – subesophageal ganglion, wlg – walking leg ganglion, wln – walking leg neuromere

**Fig. 6:**
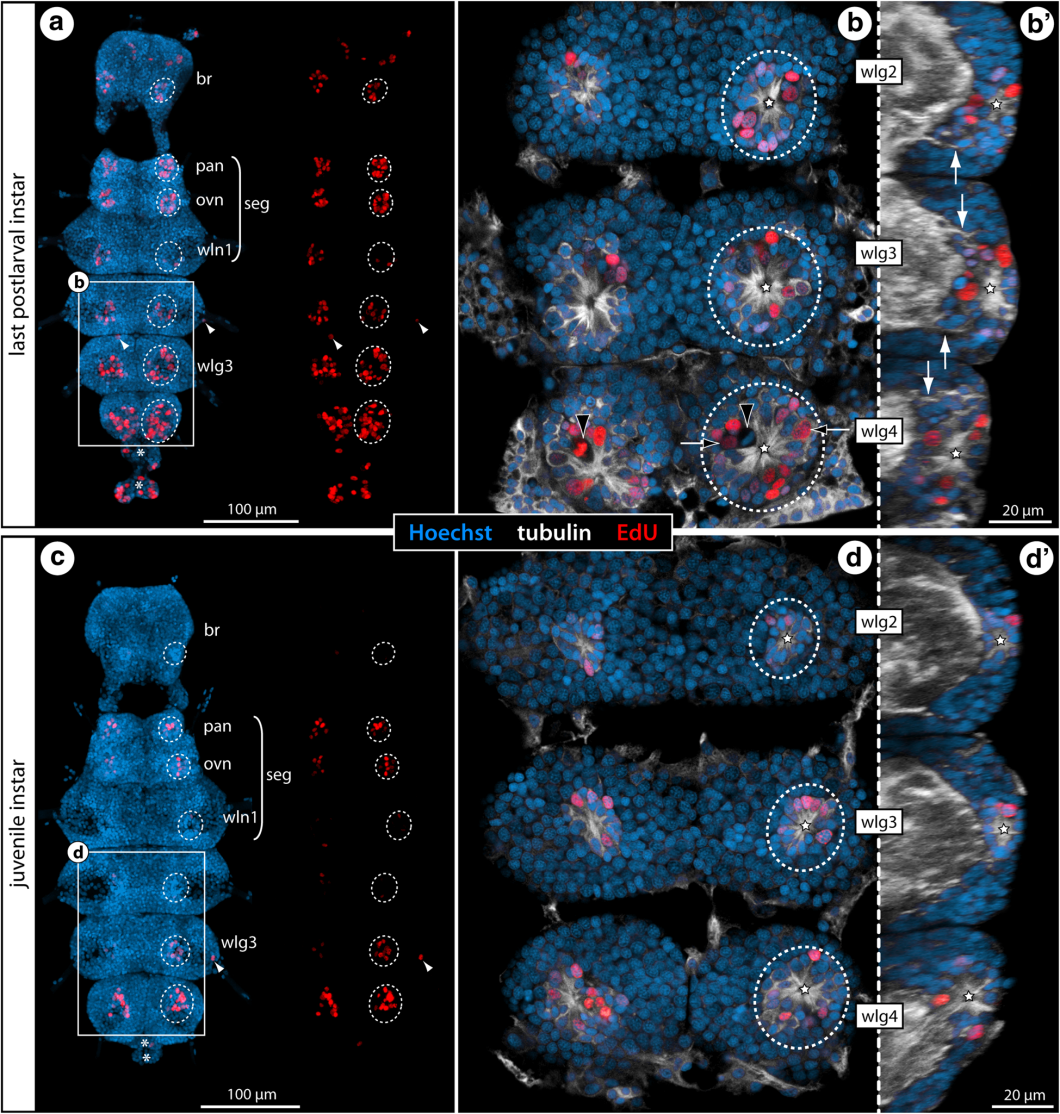
Cell proliferation in the VNC of postlarval and juvenile *T. orbiculare* instars. Labeling of acetylated tubulin (white) and EdU (red) with nuclear counterstain (blue) after 4 h EdU exposure. Maximum projections (**a**, **c**) and optical sections (**b**, **b’**, **d**, **d’**) of wholemount CNSs, anterior to the top. Stippled ovals highlight the internal VOs of one body half. Stars indicate the central cavity around which tubulin-rich cell processes converge. White arrows indicate broad tubulin-rich cell bands and condensed fibrous strands extending dorsally from the VOs towards the neuropil. White arrowheads mark single EdU-positive cells in the periphery of the ganglia. Asterisks highlight transient posterior ganglion anlagen. **a**: CNS of last postlarval instar, ventral view, EdU labeling shown separately on the right. Note high cell proliferation in palpal and ovigeral VOs as compared to the posteriorly adjacent VOs. **b** & **b’**: Wlg2–4, horizontal and sagittal sections. Black arrows highlight selected larger nuclei in the VOs of wlg4. Black arrowheads mark mitotic cells with asymmetrically positioned metaphase plates. **c**: CNS of the first juvenile instar, ventral view, EdU labeling shown separately on the right. **d** & **d’**: Wlg2–4, horizontal and sagittal sections. Only few large VO nuclei remain, VO nuclei being slightly smaller than the neuronal nuclei in the soma cortex. Abbreviations: br – brain, ovn – ovigeral neuromere, pan – palpal neuromere, seg – subesophageal ganglion, wlg – walking leg ganglion, wln – walking leg neuromere

**Fig. 7:**
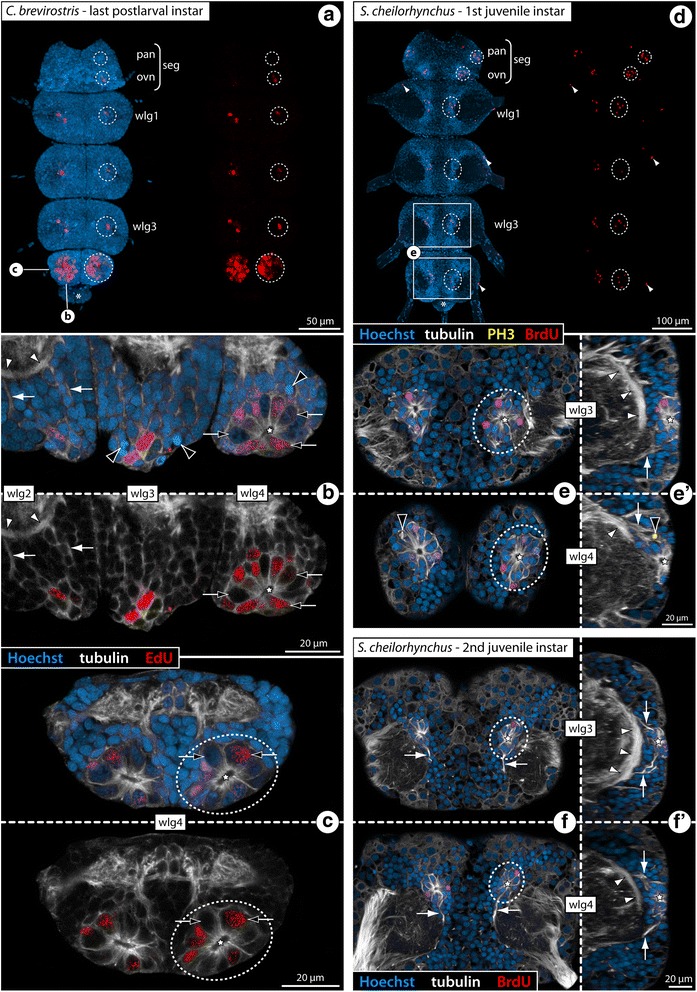
Cell proliferation in the VNC of *C. brevirostris* and *S. cheilorhynchus* instars. Labeling of acetylated tubulin (white) with nuclear counterstain (blue). *C. brevirostris*: 4 h EdU (red, **a**–**c**). *S. cheilorhynchus*: 12 h BrdU + 12 h sea water (red, **d**–**f’**). Maximum projections (**a**, **d**) and optical sections (**b**, **c**, **e–f’**) of wholemount VNCs. Stippled ovals highlight internal VOs of one body half. Stars indicate cavities around which tubulin-rich cell processes converge. Black arrows highlight larger nuclei in the VO of wlg4. White arrows indicate cell bands and condensed fibrous strands extending dorsally from the VOs towards the neuropil. Asterisks mark transient posterior ganglion anlagen. **a**: VNC of last postlarval instar, ventral view, EdU signal shown separately on the right. Note conspicuously higher number of EdU+ cells in wlg4. **b**: Wlg2–4, sagittal section. Black arrowheads mark potential pycnotic bodies indicative of cell death occurring in addition to cell proliferation. White arrowheads mark the ventral longitudinal tract **c**: Wlg4, cross section. **d**: VNC of first juvenile instar, ventral view, BrdU labeling shown separately on the right. White arrowheads mark single BrdU-positive cells in the periphery of the ganglia. **e** & **e’**: Wlg3–4 of first juvenile instar, horizontal and sagittal sections. Black arrowhead indicates a PH3-labeled (yellow) mitotic cell. White arrowheads mark ventral longitudinal tracts. **f** & **f’**: Wlg3–4 of second juvenile instar, horizontal and sagittal sections. White arrowheads mark ventral longitudinal tracts. Abbreviations: ovn – ovigeral neuromere, pan – palpal neuromere, seg – subesophageal ganglion, wlg – walking leg ganglion, wln – walking leg neuromere

For *Ph. femoratum*, *T. orbiculare* and *C. brevirostris*, the last postlarval instars were available. Additionally, juvenile instars could be studied in *Ph. femoratum* and *T. orbiculare* (Table 1). Owing to the comparably fast postembryonic development of the three species (see [33, 44]), we exposed specimens to EdU for only 4 h prior to sacrifice. In all three species, this relatively short time window proved sufficient to detect significant numbers of EdU+ cells in the CNS. Almost all of the EdU+ cells are concentrated in paired, segmentally arrayed regions in the VNC ganglia as well as in the brain (Figs. 5a, c, 6a, c and 7a; Additional files 1, 3). Furthermore, few single EdU+ cells are found in deeper layers of the cortex (Additional file 1) or are scattered more peripherally, often directly underlying the neural sheath between two adjacent ganglia or close to the segmental nerve roots (Figs. 5c and 6a, c). The peripheral location and the frequently flattened nuclei suggest a glial nature of most of these scattered EdU+ cells.

**Table 1:** Overview of species, postembryonic stages and number of specimens analyzed in the course of this study

Species	Family	# scanned & analyzed specimens			
		penultimate postlarval instar	last postlarval instar	1st juvenile instar	later juvenile instar
*Phoxichilidium femoratum* (Rathke, 1799)	Phoxichilidiidae	1	31	12	–
*Tanystylum orbiculare* Wilson, 1878	Ammotheidae	4	5	4	1
*Meridionale* sp. (“variabilis” complex sensu Arango & Brenneis, 2013 [45])	Callipallenidae	–	–	–	6
*Callipallene brevirostris* (Johnston, 1837)	Callipallenidae	–	6	–	–
*Stylopallene cheilorhynchus* Clark, 1963	Callipallenidae	–	–	5	1
*Pycnogonum litorale* Strøm, 1762	Pycnogonidae	8	5	1	–

In order to further characterize the paired ganglionic regions with the concentrated cell proliferation, EdU detection was coupled with tubulin immunolabeling. The EdU+ cells are located ventrally in the soma cortex, show more intense tubulin labeling than the surrounding somata and are arranged in conspicuous formations of spherical to ellipsoid shape. Within these formations, centrally directed processes of the intrinsic cells converge around a tiny cavity (Figs. 5b, b’, d, d’, 6b, b’, d, d’ and 7b, c; Additional file 1). This specific cell arrangement identifies these structures as the internal VOs that have been previously reported in some pycnogonids [33, 36, 38] and is strikingly similar to the arrangement in the external VOs of *Meridionale* sp. ([19, 22], see above). In further correspondence to the external VOs, the internal VOs undergo characteristic changes during development. The older a developing ganglion is, the more compact the internal VO that houses the EdU+ cells tends to be (Figs. 5b, d, 6b, d and 7b). Early stages of the internal VOs encompass many cells that feature a large nucleus with euchromatic DNA (judging from staining intensity), being distinctly larger than the nuclei of adjacent VO cells and the nuclei of cells in the surrounding cortex (e.g., Additional file 1). In contrast, the nuclei in more advanced (i.e., older) internal VOs are smaller and more densely packed than the ones in the surrounding cortical regions (Figs. 5b, d, 6b, d and 7b, c; Additional file 4). In accordance with the age-related changes in nuclear morphology, the number of EdU+ cells changes over time. In the ganglia of postlarval instars, higher numbers of EdU+ nuclei are found than in the ganglia of the older juvenile instars (Figs. 5a, c, 6a, c and 7a). This suggests a decrease in cell production with ongoing postembryonic development. Similarly, the intra-individual developmental gradient is reflected in higher numbers of EdU+ cells in the more posterior walking leg ganglia with their more voluminous VOs. Interestingly, while this gradient along the VNC appears rather linear in the postlarval instars of *Ph. femoratum* and *T. orbiculare* (Figs. 5a and 6a), a sharp increase in EdU+ cells is detectable between walking leg ganglia 3 and 4 of *C. brevirostris* (Fig. 7a, b). Further, the internal VOs of the palpal and ovigeral neuromeres in *Ph. femoratum* and *T. orbiculare* break the pattern, since they continue to exhibit comparably high cell proliferation in juveniles, even as the numbers of EdU+ cells in the posteriorly adjacent walking leg neuromeres decrease (Figs. 5c and 6c).

In contrast to the external VO-clusters of *Meridionale* sp., the internal VOs are not anatomically distinct from the surrounding soma cortex and any directed movement of cells between VOs and the cortex is more challenging to reconstruct in fixed samples (see below for *T. orbiculare*). However, it is interesting to note that anterior and posterior cell bands extend dorsally from each VO through the soma cortex towards the ganglionic neuropil, similar to the cell streams found in *Meridionale* sp. [19, 22]. While these strongly tubulin-positive cell bands are still rather difficult to delimit from the surrounding cells in earlier stages, they condense to more compact strands with accompanying small nuclei in later stages (Figs. 5b’, 6b’ and 7b; Additional files 1, 4). These strands are positioned roughly at the same level as the prominent ventral longitudinal tract that encircles the neuropil.

In *S. cheilorhynchus*, only first juvenile instars and a single specimen of the second juvenile instar were available for in vivo cell proliferation studies (12 h BrdU, 12 h sea water, sacrifice) (Table 1). The general findings for this callipallenid species are identical to the other three species (Fig. 7d–f’) including well-defined fibrous strands emanating from the compact internal VOs into the surrounding cortical areas (Fig. 7e’–f’). In line with the juveniles of the other species, the developmental gradient along the a-p axis is not as pronounced in the first and second juvenile instars of *S. cheilorhynchus*.

**Fig. 8:**
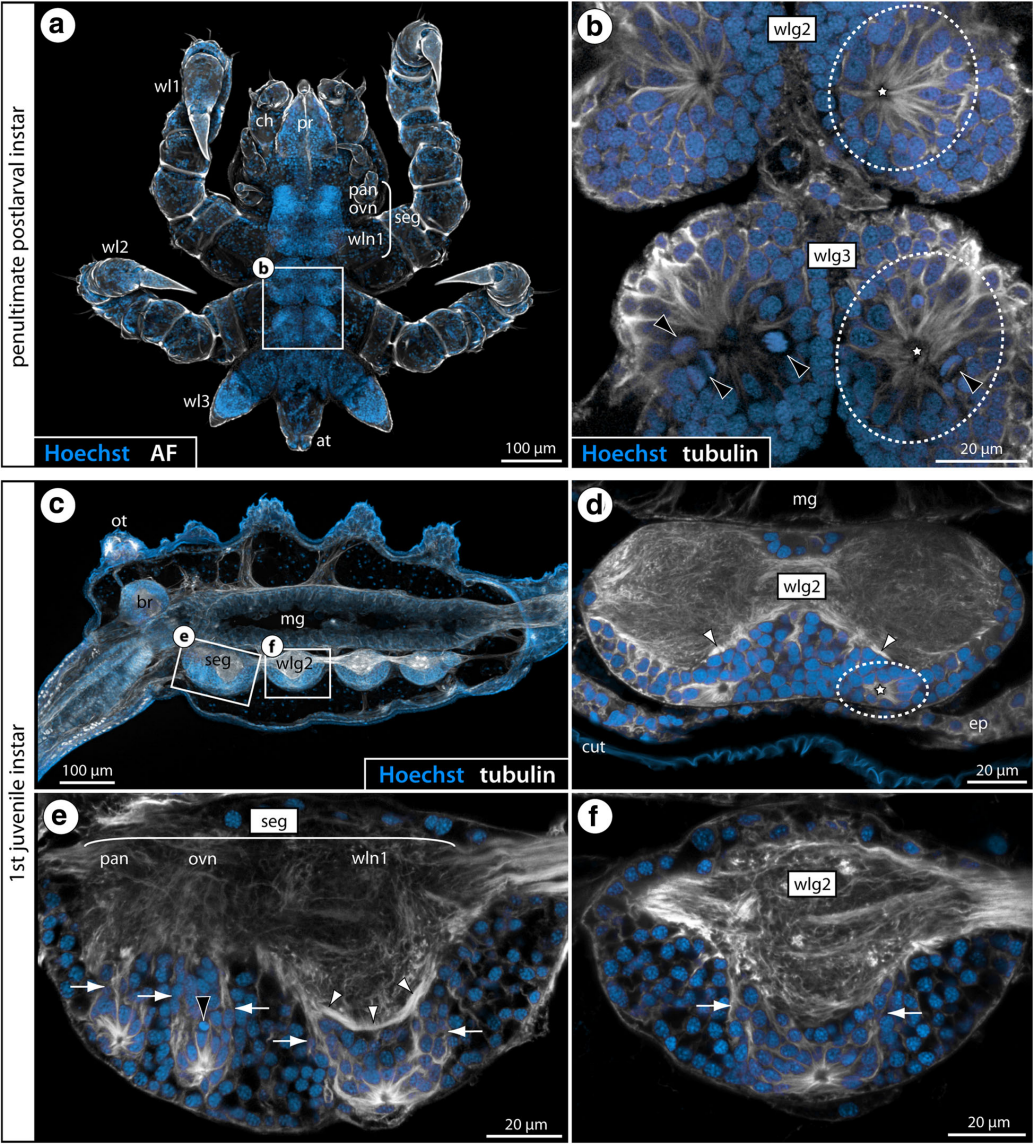
Ventral organs in the VNC of postlarval and juvenile *P. litorale* instars. Acetylated tubulin labeling shown in white – except for (**a**), which depicts cuticular autofluorescence in white. Nuclear counterstaining shown in blue. Maximum projections (**a**, **c**) and optical sections (**b**, **d**–**f**). Stippled ovals highlight the internal VOs of one body half. Stars indicate the central cavity around which tubulin-rich cell processes converge. White arrows indicate broad tubulin-rich cell bands and fibrous strands extending dorsally from the VOs towards the neuropil. White arrowheads mark the ventral longitudinal tracts. **a** Ventral overview of complete penultimate postlarval instar. **b** Wlg2–3, horizontal section. Black arrowheads highlight several mitoses in the VOs of wlg3. **c** First juvenile instar, sagittal vibratome section. **d** Wlg2, cross section. **e** Subesophageal ganglion, sagittal section. Black arrowhead marks potential pycnotic body indicative of cell death occurring in addition to cell proliferation. **f** Wlg2, sagittal section. Abbreviations: br – brain, cut – cuticle, ep – epidermis, mg – midgut, ot – ocular tubercle, ovn – ovigeral neuromere, pan – palpal neuromere, seg – subesophageal ganglion, wlg – walking leg ganglion, wln – walking leg neuromere

In *P. litorale*, no in vivo cell proliferation markers could be used owing to the lack of live postembryonic instars at the time of the experiments. However, tubulin immunolabeling coupled with nuclear counterstaining demonstrates similar internal VOs in postlarval and juvenile instars of this species (Fig. 8). In particular in the first juvenile instar, the VOs with their centrally converging cell processes and the dorsally emanating cell bands are easily discernible in the soma cortex of the ventral ganglia (Fig. 8c–f).

### Asymmetric divisions in internal VOs and first insights into the duration of the S/G_2_/M phase

Recent investigations on the neurogenic processes in *Meridionale* sp. [19, 22] have supported classical histological works on pycnogonid development that claimed a neural stem cell (NSC)-like precursor type to reside in the segmental VOs of pycnogonids [33, 36, 39]. After confirming the presence of the internal VOs, we further scrutinized them in three of the species (*Ph. femoratum*, *C. brevirostris*, *T. orbiculare*) to elucidate whether specific cell types can be morphologically distinguished and to gain more insights into their cell cycle and cell division characteristics.

**Fig. 9:**
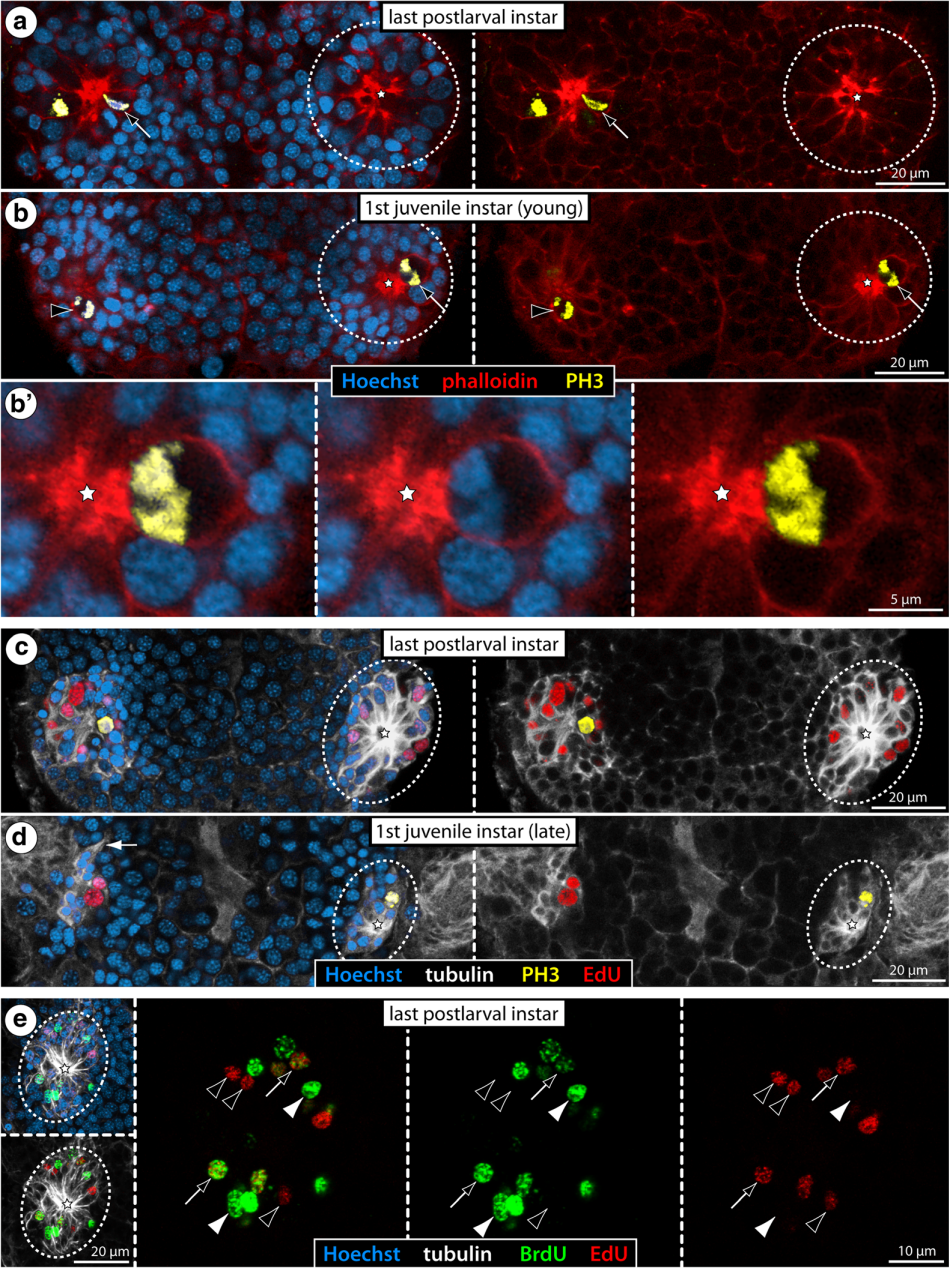
Asymmetric divisions, cell cycle aspects and BrdU clearing time of VO cells in *Ph. femoratum*. **a**, **c**: Walking leg ganglion 4 of last postlarval instar. **b**, **b’**, **d**: Walking leg ganglion 4 of first juvenile instar. Stippled ovals highlight the internal VOs of one body half. Stars indicate the central cavity around which tubulin-rich cell processes converge. **a**, **b**: Horizontal optical sections showing phalloidin-F-actin staining (red) in combination with PH3 labeling (yellow) and nuclear counterstain (blue). Black arrows point to mitotic cells with asymmetrically positioned metaphase plates. Black arrowhead marks later stage of an asymmetric division. **b’**: Higher magnification of a PH3-labeled metaphase plate with distinct shift towards one pole of the cell (the same metaphase is highlighted in **b**). **c**, **d**: Horizontal optical sections showing EdU staining (4 h exposure, red) in combination with acetylated tubulin (white) and PH3 (yellow) labeling and nuclear counterstain (blue). The PH3-positive mitotic cells show no co-labeling for EdU. The white arrow indicates the anterior fibrous strand extending dorsally from the VO. **e**: VO of last postlarval instar, extended optical section (5 μm) through one hemisphere of walking leg ganglion 3 after double-nucleoside labeling (6 h BrdU, 12 h sea water, 3 h EdU, sacrifice). Detection of BrdU (green) and EdU (red) combined with acetylated tubulin (white) and nuclear counterstain (blue). Black arrows mark selected BrdU+/EdU+ nuclei. White arrowheads highlight some BrdU+/EdU− nuclei. Black arrowheads indicate BrdU−/EdU+ nuclei

In *Ph. femoratum* (for which the highest number of specimens was available, see Table 1), we performed phalloidin labeling of F-actin to visualize the cortical cytoskeleton in combination with immunolabeling of the mitosis marker phosphorylated histone H3 (PH3) on the last postlarval and first juvenile instars. In both instars, this staining revealed mitoses in the internal VOs (Fig. 9a–b’), predominantly in the central area housing the larger cells with euchromatic nuclei. Starting in metaphase, mitoses of these cells were observed to be distinctly asymmetric, the metaphase plate being shifted far towards one pole of the cell (Fig. 9a–b’). Also in the subsequent mitotic phases this asymmetry remains discernible (Fig. 9b). This observation provides first morphological evidence for the presence of an asymmetrically dividing NSC-like precursor type in the VOs of *Ph. femoratum*. In other postlarval and juvenile specimens, we combined EdU labeling (4 h exposure, sacrifice) with subsequent immunolabeling against PH3. The EdU+ cells within the VOs were found to be significantly more numerous than the PH3+ cells, especially in the postlarval instars (Fig. 9c). This is not surprising given that PH3 is found at detectable levels only in cells that undergo mitosis (M phase) at the moment of tissue fixation, as opposed to the prolonged EdU incorporation over a time period of 4 h during which more cells may start to replicate their DNA (S phase). Interestingly, however, none of the PH3+ cells showed co-labeling with EdU (Fig. 9c, d), indicating that S phase and M phase in the proliferating VO cells are separated by at least 4 h in the *Ph. femoratum* instars.

**Fig. 10:**
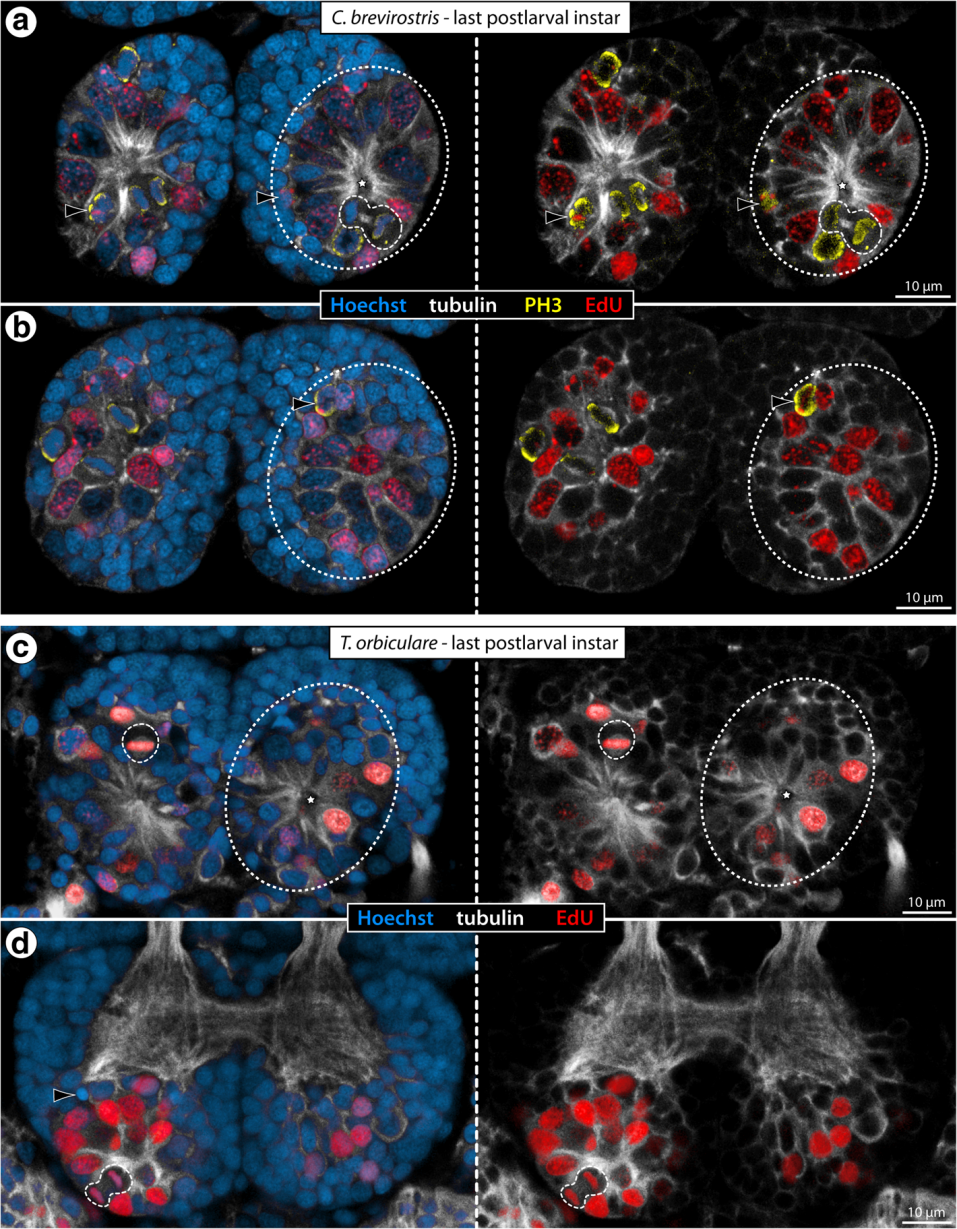
Asymmetric divisions and cell cycle aspects of VO cells of *C. brevirostris* and *T. orbiculare*. Horizontal optical sections through walking leg ganglion 4 of the last postlarval instars of *C. brevirostris* (**a**, **b**) and *T. orbiculare* (**c**, **d**). EdU labeling (4 h exposure, red) coupled to acetylated tubulin (white) and nuclear counterstain (blue). PH3 labeling (**a**, **b**) shown in yellow. Stippled ovals highlight the internal VOs of one body half. Stars indicate the central cavity around which tubulin-rich cell processes converge. **a**, **b** Advanced mitotic stages (=metaphase and later) of the VO cells of *C. brevirostris* show no EdU co-labeling, whereas earlier mitotic phases have weak EdU signal (black arrowheads). Note the asymmetry in the division of one large neural precursor cell (stippled outline). **c**, **d** Mitotic VO cells in metaphase and even telophase are EdU-positive and show indications of morphologically asymmetric divisions (stippled outlines). The black arrowhead marks a potential pycnotic body indicative of cell death occurring in addition to cell proliferation

The same EdU/PH3 labeling approach was applied to the few available postlarval instars of *C. brevirostris* and gave similar results. At this stage, the highest cell proliferation occurs in the VOs of walking leg ganglion 4 (Fig. 7a). As in *Ph. femoratum*, the EdU+ cells distinctly outnumber the PH3+ cells (Fig. 10a, b). Yet, while advanced mitotic stages were found to be EdU-negative, some PH3+ cells in prophase showed weak EdU co-labeling (Fig. 10a, b). This observation speaks for a shorter G_2_ phase between the S and M phases in the large proliferating VO cells and may indicate a generally faster cycling compared to *Ph. femoratum*. Close inspection of later mitotic stages of these large cells also revealed a slight asymmetry in the divisions (Fig. 10a), which is in agreement with previous claims of NSC-like precursors in the VOs of *C. brevirostris* [33].

In *T. orbiculare*, the VOs of the last postlarval instar were studied after EdU exposure (4 h exposure, sacrifice). Not all of the EdU+ cells in the VOs show notable size differences compared to the somata in the surrounding cortex (Fig. 10c, d). Nonetheless, several of the observed mitotic stages in larger cells show the same indications of morphological asymmetry with shifted metaphase plates (Figs. 6b and 10c) and persisting slight differences in later mitotic stages (Fig. 10d). Notably, very strong EdU labeling was encountered in cells in all mitotic phases, including the telophase (Fig. 10c, d). At the same time, however, some mitotic cells were devoid of EdU labeling (Fig. 6b). Accordingly, the cycling times of the proliferating VO cells in *T. orbiculare* seem to be more heterogeneous, comprising faster (S phase to M phase < 4 h) and slower (S phase to M phase > 4 h) cycling cells. With the available data it cannot be determined whether these cell cycle differences relate to different neural precursor types in the VOs.

### Double-nucleoside labeling using BrdU and EdU – estimation of BrdU clearing time and further evidence for NSC-like precursor types in pycnogonid VOs

During the postembryonic development of *Meridionale* sp., the direction of cell movement from external VO-clusters via the streams into the soma cortex has been previously reconstructed [22] and independently validated here with double-nucleoside cell proliferation experiments (Fig. 4; Additional file 2). Aiming to investigate whether cell migration occurs in species with internal VOs, we conducted similar experiments with postembryonic instars of *Ph. femoratum* and *T. orbiculare*. Experiments with postlarval instars and juveniles of *T. orbiculare* were successful and lasted 3 days in total (see next section). In *Ph. femoratum*, by contrast, survival of postembryonic instars in good condition could only be accomplished with short time windows of less than 24 h. This duration was too short to extract cell migration patterns with any confidence (Additional file 4). However, although the experiments failed in this respect, the relatively short time intervals between the two proliferation marker pulses provided insights into the marker clearing time in pycnogonid instars falling in the studied size range: The shortest applied sea water interval between BrdU and EdU exposures was 12 h. After this experiment, the VOs of *Ph. femoratum* showed a mixed pattern of BrdU+/EdU- and BrdU+/EdU+ cells, but importantly also some BrdU-/EdU+ cells (Fig. 9e; Additional file 4). The presence of EdU-only labeled cells indicates that BrdU was no longer available at detectable levels after the intervening 12 h in sea water, resulting in the exclusive incorporation of EdU in cells passing through S phase during the EdU exposure period. Accordingly, 12 h can be deduced as a conservative estimate for the clearing time of BrdU from the animals. This may still be an overestimation since shorter inter-pulse intervals have not been tested.

Regardless of the uncertainty pertaining to shorter inter-pulse intervals, this finding has important implications for experiments using longer inter-pulse intervals with the similar-sized postembryonic instars of *Meridionale* sp. The intervening sea water interval in our double-labeling experiments on *Meridionale* sp. juveniles lasted almost 4 days (92 h), that is, almost 8-times the 12 h clearing time established in *Ph. femoratum*. Therefore, it seems highly unlikely that any of the double-labeled cells observed in *Meridionale* sp. (Fig. 4b–e; Additional file 2) incorporated BrdU during the EdU exposure period. As a consequence, double labeling with both proliferation markers suggests that cells have repeatedly passed through S phase during the experiment. Such double-labeled cells are almost exclusively found in the external VO-clusters of *Meridionale* sp. and only occasionally in the proximal portions of the migratory streams (Fig. 4b–e; Additional file 2). Further, although BrdU-only labeled cells were readily identifiable in the external VO-clusters and deep within the soma cortex, EdU labeling is detected in the overwhelming majority of cases in combination with BrdU labeling (381 out of 383 EdU+ cells [= 99.5%] with BrdU co-labeling in six specimens). This pattern strongly suggests that repeatedly dividing neural precursor cells reside within the external VO-clusters, their progeny migrating through the streams into the cortex. Morphological delimitation of different cell types is not possible in the external VOs of juvenile instars of *Meridionale* sp. [22]. However, since the external clusters are derivatives of the earlier VO-stages that house large asymmetrically dividing NSC-like precursors [19], it is feasible that the double-labeled cells are of the same type, having decreased in size with ongoing cycling during postembryonic development.

### Double-nucleoside labeling in *T. orbiculare*: cell migration from the VOs into the soma cortex

In *T. orbiculare*, 3-day-long double-nucleoside labeling experiments were conducted (4 h BrdU exposure, 64 h sea water, 4 h EdU exposure, sacrifice) with the penultimate and last postlarval instars as well as with the first juvenile instar. Investigation of this developmental series enabled us to evaluate labeling patterns intra-individually along the a-p developmental gradient but also between specific segmental ganglia across different instars.

**Fig. 11:**
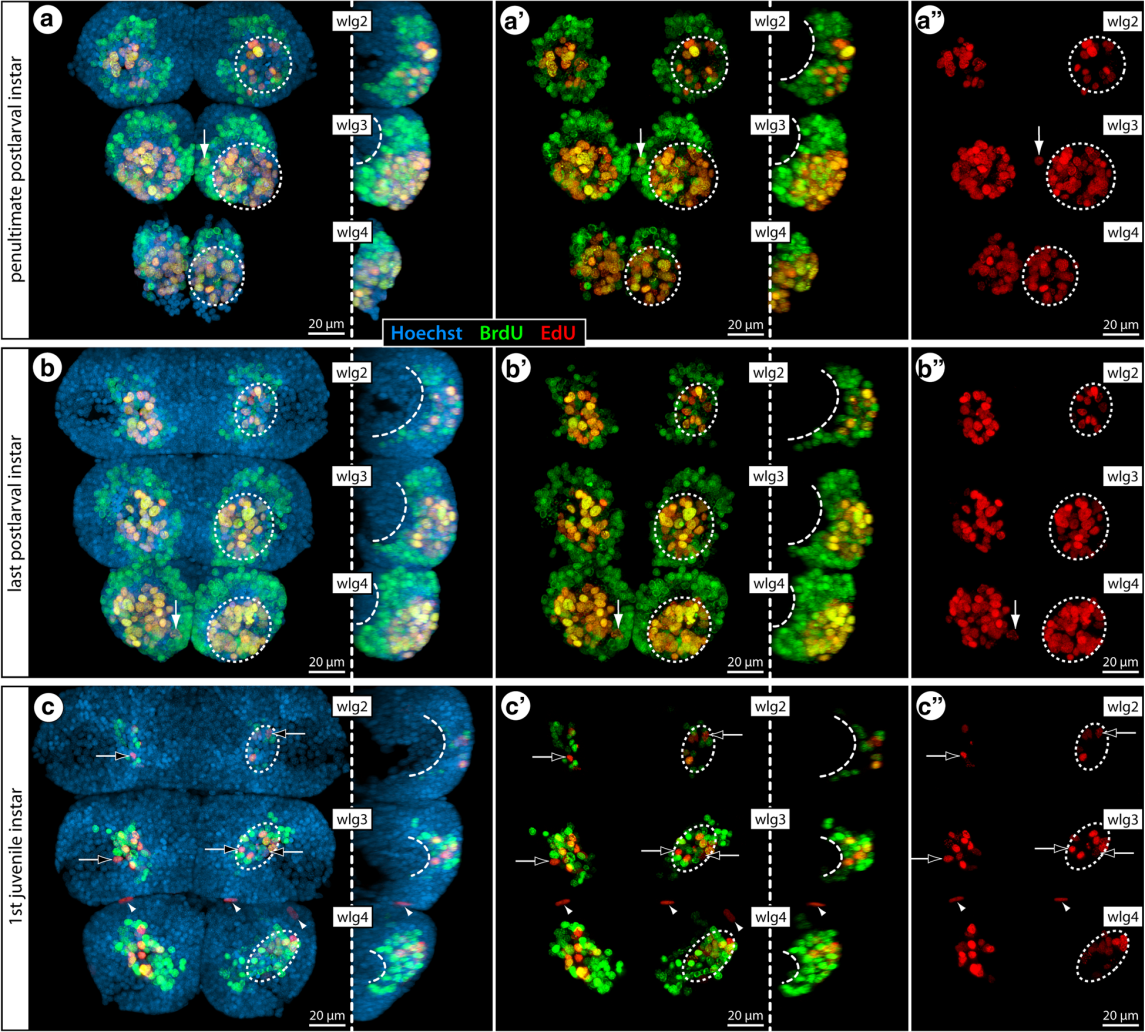
Double-nucleoside labeling experiments with postlarval and juvenile instars of *T. orbiculare*. BrdU (green) and EdU (red) labeling (4 h Brdu, 64 h sea water, 4 h EdU, sacrifice) with nuclear counterstain (blue). All images show maximum projections in ventral view, apart from those positioned to the right of stippled vertical lines, which are lateral views of the hemiganglia of one body half. For a clearer depiction of the proliferation marker patterns, the nuclear counterstain and BrdU signal have been successively removed from left to right. Stippled ovals highlight the position of the internal VOs in one body half. Curved stippled lines highlight the characteristic sickle-like arrangement of the BrdU-positive cells. Black arrows point at exclusively EdU-labeled nuclei in advanced VO-stages. White arrowheads indicate exclusively EdU-labeled, flattened nuclei of peripheral glial cells. White arrows mark selected EdU-positive nuclei of cells just outside the VOs, potentially indicative of further divisions after cells have started to migrate. **a–a”**: Wlg 2–4 of the penultimate postlarval instar. **b–b”**: Wlg 2–4 of the last postlarval instar. **c–c”**: Wlg 2–4 of the first juvenile instar. Abbreviations: wlg – walking leg ganglion

**Fig. 12:**
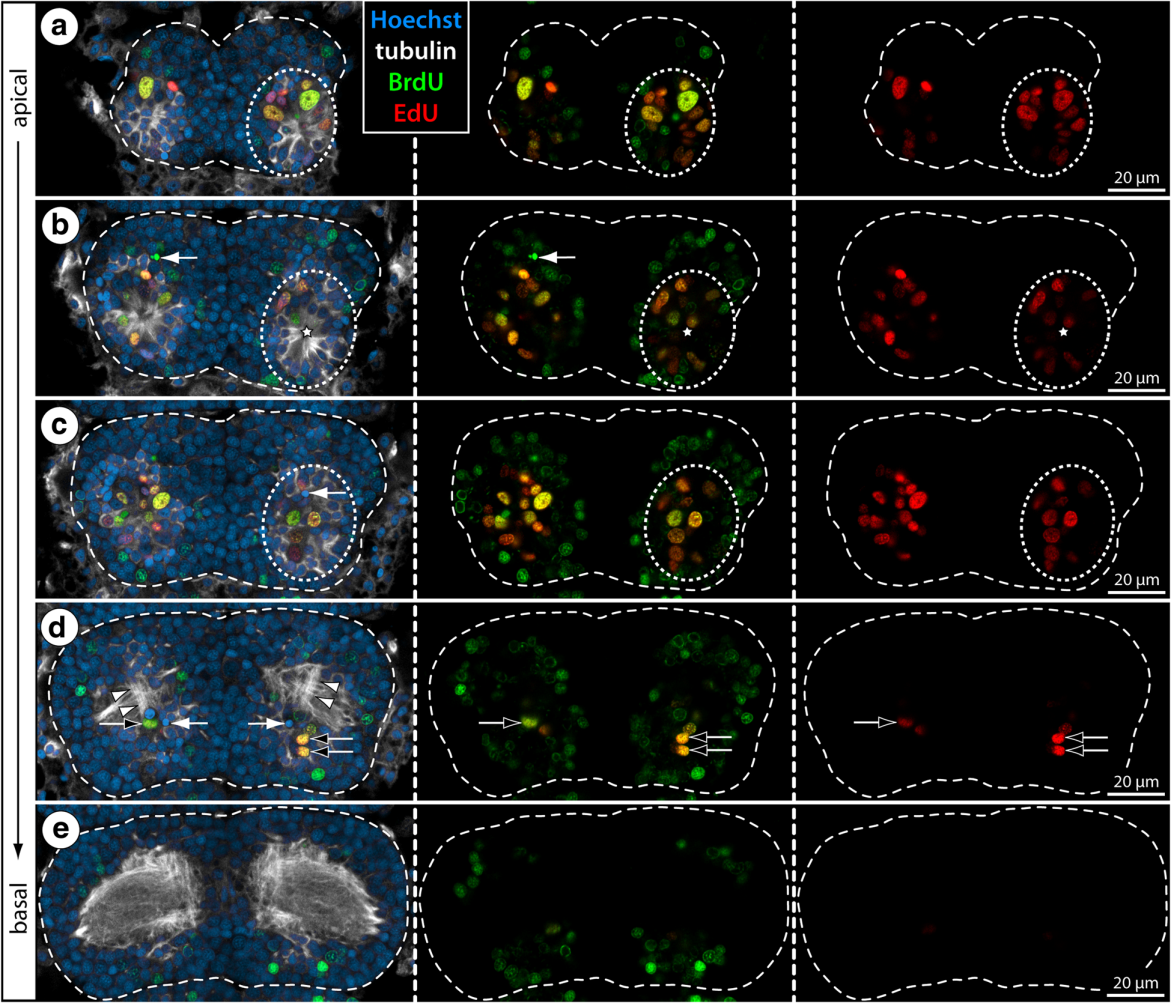
Distribution of BrdU- and EdU-positive cells in walking leg ganglion 3 of *T. orbiculare*. BrdU (green) and EdU (red) labeling (4 h Brdu, 64 h sea water, 4 h EdU, sacrifice) with acetylated tubulin (white) and nuclear counterstain (blue). All images show horizontal optical sections through walking leg ganglion 3 of the last postlarval instar, proceeding from apical to basal. For clearer depiction of the proliferation marker patterns, tubulin labeling plus nuclear counterstain and the BrdU signal have been successively removed from left to right. The wide stippled outlines trace the extension of the ganglionic soma cortex. Stippled ovals highlight the position of the internal VOs in one body half (**a**–**c**). Black arrows (**d**) point at BrdU and EdU co-labeled nuclei in the cell band emanating dorsally from the VO and running posterior to the neuropil, suggestive of further divisions during cell migration. White arrowheads (**d**) mark neurite bundles of the ventral longitudinal tract which becomes recognizable in these stages. White arrows (**b**–**d**) point at potential pycnotic bodies indicative of cell death occurring in addition to cell proliferation in the VOs and the migratory cell bands. Note the heterogeneous nucleus sizes in the internal VOs (**a**–**c**) as well as similar nucleus size and morphology of BrdU-positive cells and surrounding somata in areas outside the VOs and deeper in the cortex (**c**–**e**)

The overall patterns across instars and along the a-p gradient show that the highest cell proliferation correlates well with the major growth periods of the different ventral ganglia, being followed by a notable decrease of proliferation rates in later stages (Fig. 11). Without exception, the number of cells labeling for the first marker BrdU significantly exceeds the second marker EdU (Figs. 11 and 12; Additional file 3). Cells that have undergone S phase during the 4 h EdU pulse are predominantly restricted to the ventral soma cortex, being located in the internal VOs (Figs. 6 and 12). During the major growth period of the ganglia, the VOs house EdU+ cells of different cell sizes (Fig. 12a–c), which might point to the presence of different neural precursor cell types/generations during the peak of cell proliferation activity. Irrespective of size differences, however, almost all EdU+ cells show also BrdU co-labeling (Figs. 11 and 12; Additional file 3). However, in more advanced VO-stages, cells labeled exclusively with EdU are more frequently detectable (Fig. 11c–c”). The most obvious EdU-only cells are located in the periphery of the ganglia of juvenile instars, featuring flattened nuclei that suggest a glial nature (Fig. 11c–c”). Even though few in number, the presence of these BrdU-/EdU+ cells confirms that unbound BrdU was not available during the final EdU pulse, as predicted by the clearing time estimate obtained in *Ph. femoratum*. Consequently, the double-labeling of cells in the VOs of *T. orbiculare* indicates their repeated passage through S phase during the experiments, in accordance with the results obtained for the external VO-clusters of *Meridionale* sp.

In contrast to the EdU labeling, BrdU+ cells are spread far beyond the VOs into the growing soma cortex of the developing ganglia. They do not only surround the VOs but also reach further dorsally towards the forming ganglionic neuropil. Especially in advanced stages of ganglion development, the BrdU+ cells extend anteriorly and posteriorly from the neuropil, leading in lateral view to a sickle-like arrangement embedded in the cortex (Fig. 11a, a’, b, b’, c, c’; Additional file 3). This is strongly suggestive of active cell migration. Also in ventral view, the overall pattern of BrdU+ cells changes over time. During the early phases of rapid ganglion growth, they are spread out in broad, almost concentric rings around the VOs (Fig. 11a, a’, b, b’) as would be expected from the migration of newborn cells in all directions from the main centers of cell proliferation. In later stages, however, the BrdU+ cells are arranged in more elongated band-like patterns with an oblique a-p axis (Fig. 11c, c’). The VOs are still in the center of these cell bands but migration of newborn cells has become restricted laterally and is instead concentrated along two pathways that encircle the central neuropil anteriorly and posteriorly, in close proximity to the ventral longitudinal tract (Additional file 3). Incidentally, already in earlier stages, cells that have recently undergone DNA synthesis (i.e., are EdU+) and have started to migrate dorsally are encountered in close vicinity to the same tract (Fig. 12d).

Importantly, the considerable number of BrdU+ cells labeled during the rapid ganglion growth may not be exclusively attributable to cell proliferation in the VOs but may in part be explained by additional cell divisions during migration. Although this aspect cannot be conclusively resolved with the methods applied here, the almost complete lack of EdU labeling in deeper layers (e.g., Fig. 12c, d) suggests that such divisions of newborn cells leaving the VOs would have to occur in early stages of migration. The majority of BrdU+ cells located deeper in the cortex are therefore more likely to be differentiating/differentiated postmitotic neural cells, which notion receives further support from their similar nuclear morphology compared to the surrounding BrdU-negative neuronal somata (Fig. 12d, e).

## Discussion

### Gross architecture of the VNC: independent inclusion of walking leg neuromere 1 into the SEG of different pycnogonid lineages

**Fig. 13:**
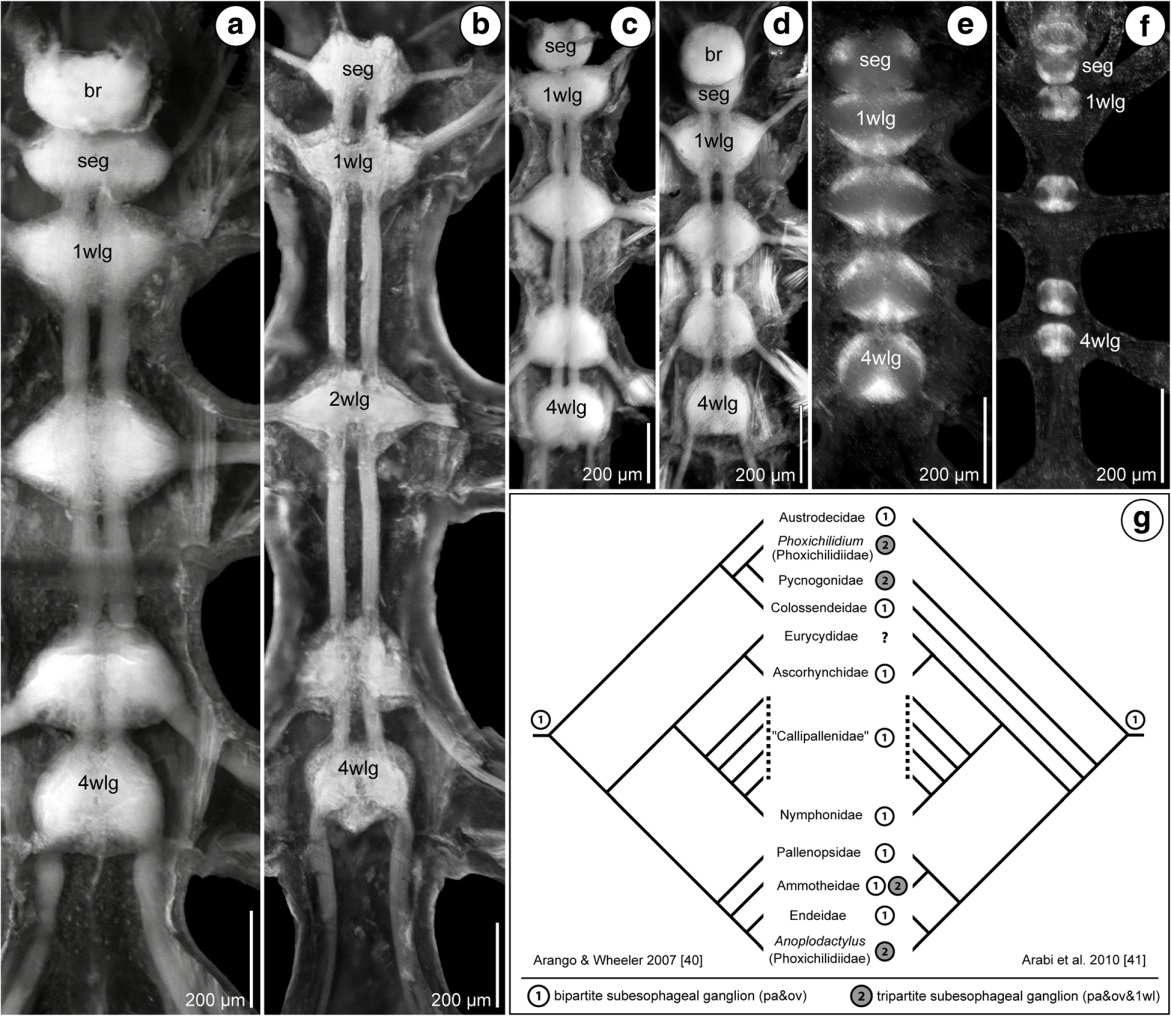
Gross architecture of the VNC of different sea spider families evaluated against the backbone of pycnogonid phylogeny. **a**–**d** Fluorescent stereomicroscope images of partially dissected nervous systems after labeling with the lipophilic marker FM 1-43FX. **e**–**f** Fluorescent stereomicroscope images of nervous systems with nuclear labeling. Note that all species feature an anatomically separate walking leg ganglion 1, even though it is always close to the subesophageal ganglion. **a** *Nymphon gracile*, adult CNS, dorsal view. **b** *Parapallene australiensis*, adult VNC, dorsal view. **c** *Endeis spinosa*, adult VNC, dorsal view. **d** *Cilunculus japonicus*, adult CNS, dorsal view. **e** *Propallene* sp., adult VNC, dorsal view. **f** *Pantopipetta armoricana*, CNS of late juvenile, ventral view. **g** The bipartite subesophageal ganglion is resolved as plesiomorphic condition of Pycnogonida after mapping on current hypotheses of pycnogonid relationships and parsimony-based reconstruction. Abbreviations: br – brain, seg – subesophageal ganglion, wlg – walking leg ganglion

In accordance with the conserved basic body organization of the various sea spider taxa (excepting the few extra-legged species), the pycnogonid CNS shows a remarkably uniform architecture [31, 32, 52]. In the majority of species, the VNC features the exact same set of separate adult ganglia, although the relative distance between them can differ significantly (Fig. 13). The latter phenomenon correlates well with the general habitus of a given species (slender, tube-like trunk vs. compact, disk-shaped trunk).

Our study highlights one of the few taxon-specific architectural differences of the pycnogonid VNC: the three callipallenid representatives possess an anatomically separate walking leg ganglion 1 posterior to a bipartite SEG that comprises the palpal and ovigeral neuromeres. By contrast, the remaining three species have a tripartite adult SEG that includes the neuromere of walking leg segment 1. Importantly, however, outside of Phoxichilidiidae, Pycnogonidae and the genus *Tanystylum,* all pycnogonid taxa hitherto studied have a bipartite SEG as found in the three callipallenids (Fig. 13a–f, also [30, 52]). This taxon list includes other ammotheid genera (e.g., Fig. 13d) as well as groups which have been indicated to branch off close to the base of the pycnogonid tree in the most exhaustive phylogenetic analyses [40, 41, 53] (Fig. 13g), such as Austrodecidae (Fig. 13f) and Colossendeidae (e.g., [31, 54]). Further, it also encompasses Ascorhynchidae (e.g., [31, 55]) which has been proposed as the earliest (paraphyletic) offshoot in one 18S rRNA-based molecular analysis [56] (but see, e.g., [53] for Ascorhynchidae well-nested in the pycnogonid tree). Hence, when mapping the SEG architecture on currently discussed topologies of pycnogonid interrelationships, a bipartite composition is resolved as the most parsimonious plesiomorphic state (Fig. 13g). The tripartite SEG of some extant taxa, on the other hand, is indicated to go back to three independent events in pycnogonid crown group evolution, being the result of the fusion of the palpal, ovigeral and walking leg 1 neuromeres during postembryonic gangliogenesis. Notably, this interpretation holds true regardless of the morphologically questionable splitting of the Phoxichilidiidae, which has been recovered due to the rather surprising placement of the genus *Phoxichilidium* in one of the recent analyses [40] (Fig. 13g).

### Variations of a common theme – external versus internal pycnogonid VOs

**Fig. 14:**
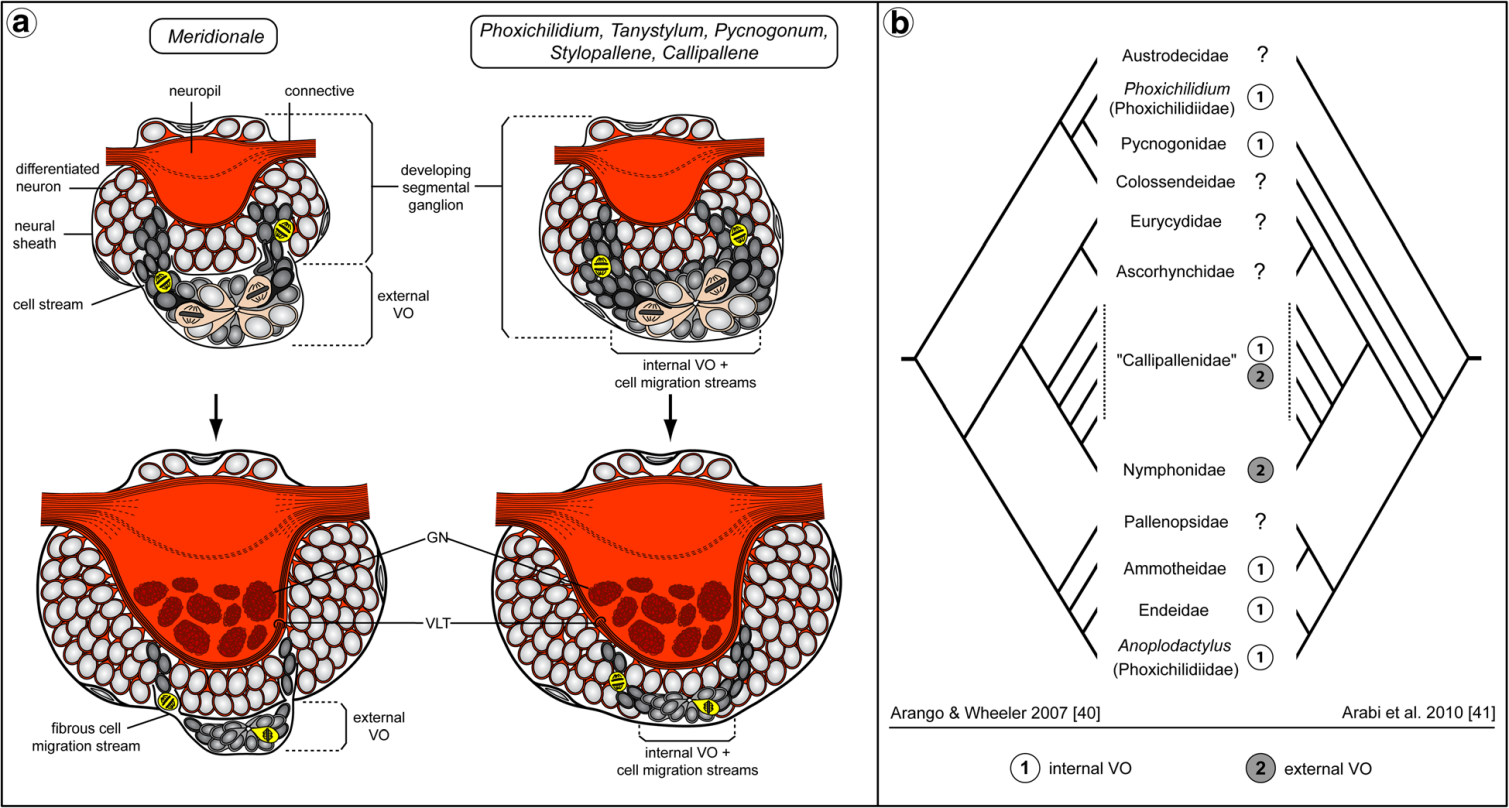
Internal versus external VOs and reconstruction of the plesiomorphic VO type in crown group Pycnogonida. **a** Schematic representation of external and internal VOs from postlarval to juvenile instars, showing an idealized sagittal section. Lightly-colored, larger cells in the VOs represent the NSC-like precursors. Black cells detach from the VOs and start migration. Yellow cells are neural precursors that divide again while they migrate into the soma cortex (as shown in [22]). **b** Mapping of the VO types on two competing hypotheses on the phylogenetic relationships of extant pycnogonid taxa. Although several lineages remain unstudied (labeled with question marks), the distribution of the two VO types across the taxa investigated suggests internal VOs to be the plesiomorphic condition of Pycnogonida. Abbreviations: GN – glomerulus-like neuropil, VLT – ventral longitudinal tract, VO – ventral organ

In the developing ventral ganglia of all pycnogonid species studied here, we could confirm the existence of segmental VOs, which appear as conspicuous paired clusters of cells with centrally directed processes converging around a small cavity. However, in contrast to *Meridionale* sp., where the VOs represent separate clusters that lie external to the ganglia, the ones in the five other species are incorporated into the ganglionic soma cortex (Fig. 14a), in which they appear as intensely tubulin-labeled cell formations with centrally directed processes. The latter finding confirms classical histological studies focusing on the VNC development in pycnogonid taxa other than Nymphonidae [33, 36–39].

The deviating position of the external and internal VOs raises the questions whether they serve the same function and if they are homologous structures across pycnogonids. With regard to the functional role, our cell proliferation experiments and mitosis marker labeling unambiguously characterize internal VOs as centers of significant cell proliferation during VNC development, in correspondence to the external VOs of *Meridionale* sp. [19, 22]. Further, regardless of VO position, our double-nucleoside labeling approach illustrates migration of significant numbers of newborn cells from the VOs into the growing soma cortex of the respective ganglion. Accordingly, this indicates that external and internal VOs act as postembryonic neurogenic niches in the ventral ganglia – and refutes previous speculations on the predominantly neurosecretory nature of the internal VOs [37, 38].

**Fig. 15:**
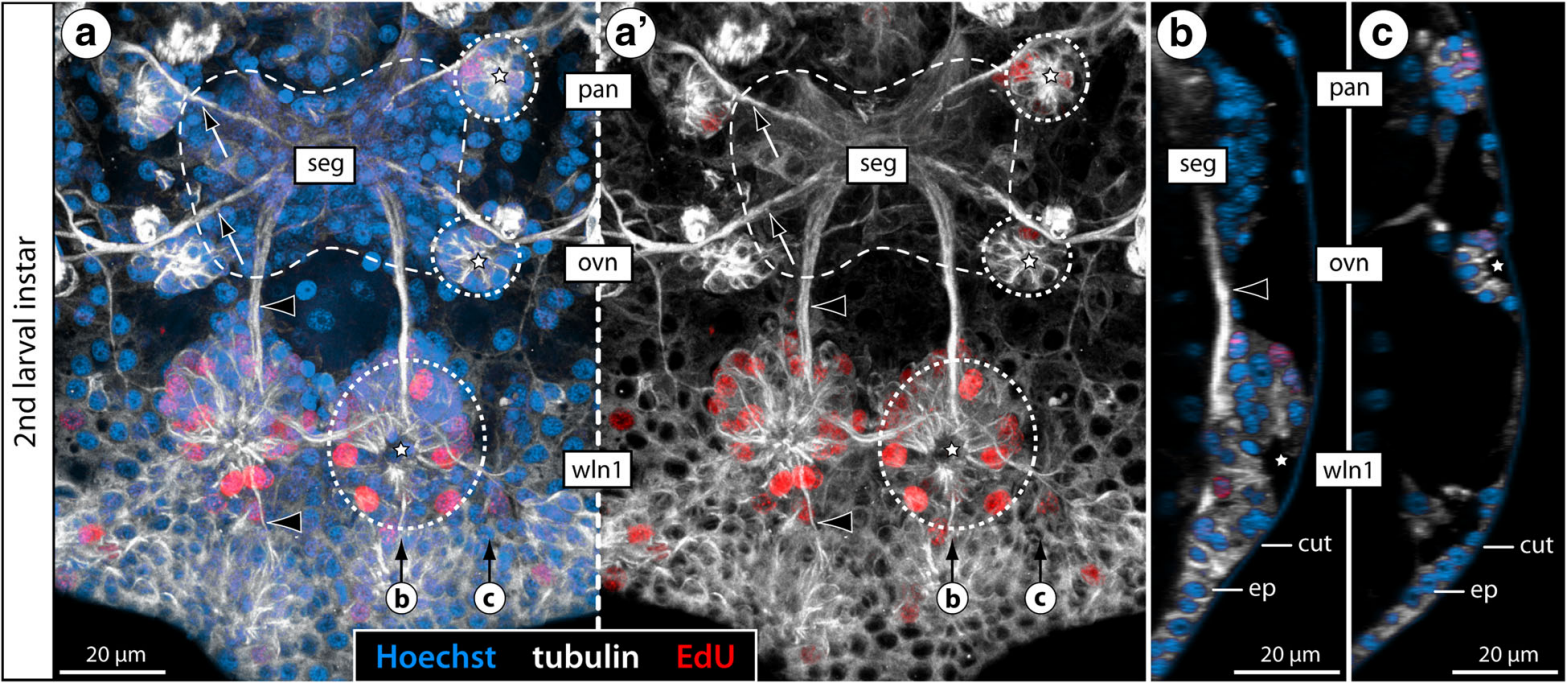
Cell proliferation in palpal and ovigeral VOs of the second larval instar of *T. orbiculare*. Labeling of acetylated tubulin (white) and EdU (4 h exposure, red) with nuclear counterstain (blue). Horizontal (**a**, **a’**) and sagittal (**b**, **c**) optical sections, anterior to the top. Stippled ovals highlight the apically attaching early VOs of one body half. Stars indicate the forming apical invagination of the early VO stages, prior to their detachment from the apical ectoderm. **a**, **a’**: Note the low number of EdU-positive cells in the palpal and ovigeral VOs as opposed to the forming VO of walking leg neuromere 1. Black arrows mark the roots of the palpal and ovigeral larval nerves as they leave the larval subesophageal ganglion. Black arrowheads highlight the first longitudinal axonal pathways of the future VNC. **b**, **c**: Note the considerable size difference between the palpal and ovigeral VOs (**c**) and the invaginating VO of walking leg neuromere 1 (**b**). Abbreviations: cut – cuticle, ep – epidermis, pan – palpal neuromere, ovn – ovigeral neuromere, seg – subesophageal ganglion, wln1 – walking leg neuromere 1

With regard to the question of homology, additional characteristics can be considered: beyond the functional correspondence of both VO types as niches of postembryonic neurogenesis, the presence of a morphologically conspicuous large cell type – especially in earlier stages – has been noted in all previous descriptions (e.g., [33, 36, 39]). In the present study, we have shown asymmetric divisions of these cells in three species with internal VOs (*Ph. femoratum*, *T. orbiculare*, *C. brevirostris*), in line with previous findings in the external VOs of *Meridionale* sp. [19]. The occurrence of such large and asymmetrically dividing cells has been recognized also in earlier VO stages of *S. cheilorhynchus* [19]. Further, our double-nucleoside labeling experiments with *T. orbiculare* have provided evidence for repeated divisions of cells residing in the internal VOs. In combination with the observed asymmetry of some divisions, these findings support the presence of a NSC-like precursor type as a shared feature of external and internal VOs (Fig. 14a). Beyond that, the developmental series of *T. orbiculare* instars investigated with double-nucleoside labeling has revealed more specific similarities to the cell migration patterns of *Meridionale* sp. ([19, 22], present study): starting with a rather homogeneous movement in dorsal direction and proceeding via two broad, antero- and posterodorsally oriented cell bands, the newborn cells become increasingly restricted to two migration streams with ongoing ganglion development. These cell migration streams extend in close vicinity to the same ventral longitudinal tract in both species (Fig. 14a), and corresponding fibrous components extending along this pathway have been detected in all other species with internal VOs. Finally, the origin and earlier developmental stages of both VO types are virtually identical. Both are derivatives of segmentally paired, pseudo-stratified regions of the ventral neuroectoderm from which the first ganglion cells immigrate (e.g., [19, 57]). With ongoing development, these regions begin to invaginate (Fig. 15a–c) and eventually detach from the apical ectoderm, thus forming the conspicuous paired VOs at the ventral side of the ganglion anlage, the VOs’ central cavities being lined by the formerly apical poles of the neuroectodermal cells [19, 22, 33, 35, 39]. Considering all of these correspondences, we find strong support for the homology of both types of VO in the various pycnogonid taxa, in spite of the positional difference. This difference is the result of an incorporation process into the soma cortex during postembryonic gangliogenesis of some species, which does not take place in others.

In an evolutionary framework, it remains then to be clarified which of the two VO types represents the plesiomorphic condition of the taxon Pycnogonida. Across the groups studied so far, external VOs have been exclusively discovered in Nymphonidae (e.g., [35, 36, 58]) and the callipallenid genera *Meridionale* ([22], present study) and *Pseudopallene* [58]. In all other taxa investigated, including the callipallenid genera *Stylopallene* and *Callipallene* ([33, 38, 39], present study), the VOs are incorporated into the soma cortex (Fig. 14b). Irrespective of which phylogenetic hypothesis is favored [40, 41, 53, 56], a parsimony-based assessment of the distribution of VO types suggests the external VOs to be an apomorphic condition that has emerged within the callipallenid-nymphonid lineage (Fig. 14b). To date, the exact relationships of the nymphonid and callipallenid taxa are still unsatisfactorily resolved. However, with further progress in this field, the presence of external VOs may prove phylogenetically informative, as a potential apomorphic character state of a monophylum comprised of a subset of callipallenid species and nymphonids.

### Interspecific differences and deviations from the a-p gradient of VO cell proliferation correlate to specific events of postembryonic development

Compared to the rather linear a-p gradient of ventral ganglion development in *Ph. femoratum* and *T. orbiculare*, the last postlarval instar of *C. brevirostris* shows an abrupt increase in cell proliferation between the VOs of walking leg ganglia 3 and 4 (Fig. 7a). This observation coincides with a different mode of postembryonic development. The genus *Tanystylum* shows a pronounced anamorphic development with a minute protonymphon larva as hatching stage and strictly sequential addition of the walking leg segments [33, 59]. This developmental type is widely considered to be plesiomorphic for Pycnogonida (e.g., [42]). Likewise, all members of the Phoxichilidiidae follow an anamorphic – albeit accelerated – mode with a slight but perceivable a-p gradient in walking leg segment formation [44, 60]. By contrast, the two genera *Callipallene* and *Neopallene* feature the most pronounced embryonization of development known in Pycnogonida [33, 38, 39, 51, 54]. One hallmark of their development is the synchronized differentiation of walking leg segments 1–3 early during embryogenesis, whereas walking leg segment 4 is formed distinctly later [42]. As a consequence, the first three walking leg segments and their ganglia are well-differentiated in the postlarval instar, but walking leg segment 4 is still in the period of major ganglion growth with VOs that house numerous large NSC-like precursors (Fig. 7b, c). Accordingly, the observed differences in overall cell proliferation patterns between postlarval instars of the different species examined reflect deviations in the timing of walking leg segment formation during their development.

Another case of proliferation patterns deviating from a monotonous a-p developmental gradient is encountered in the anterior palpal and ovigeral VOs that are affiliated with the SEG. While cell proliferation of the posteriorly following VOs in walking leg segments 1 and 2 decreases between the last postlarval and the first juvenile instar, the number of EdU+ cells in the palpal and ovigeral VOs was found to remain distinctly higher in *Ph. femoratum* and *T. orbiculare* (Figs. 5A, c and 6a, c). Again, these observations correlate with developmental events in this particular region: all pycnogonid taxa with a protonymphon larva as hatching stage possess small three-segmented palpal and ovigeral larval limb pairs at the beginning of the postlarval phase [57, 58, 61–63]. However, one of the most peculiar aspects of pycnogonid development is the metamorphosis of these limbs into the adult palps and ovigers (if present) which starts at the end of the postlarval phase. In the case of the oviger, this metamorphosis even includes the almost complete atrophy of the larval limb prior to a de novo outgrowth of the appendage during the first instars of the juvenile phase (see [59] for *Tanystylum*, [51] for review). Accordingly, major neuroarchitectural changes are to be expected in the SEG during this period, owing to the loss of neural elements of the palpal and ovigeral appendage nerves of the larva (Fig. 15a, a’) as well as the integration of new afferent and efferent neurons into the existing circuits as the adult limb pairs differentiate. Embedded in this developmental framework, the higher cell proliferation rates in the corresponding VOs appear a logical consequence, and may serve as additional evidence for the VOs' indicated neurogenic role.

### Beyond sea spiders: Internal VOs as a plesiomorphic feature of arthropod gangliogenesis

Our new data on the internal VOs in some pycnogonid taxa and the reconstruction of this VO type as the plesiomorphic condition of Pycnogonida has implications in a broader arthropod-wide framework.

Similarities and differences between neurogenic processes in the major arthropod groups and their closest relatives have been discussed in depth in numerous contributions of the last decades (e.g., [18–20]), often with focus on the initial embryonic phase and to the inclusion of the involved gene networks. In this context, the discovery of detailed similarities between the neural precursor types and neural cell lineages underlying early embryonic neurogenesis in hexapods and (some) crustaceans (see [3, 6, 18, 64]) has contributed compelling neurodevelopmental arguments in favor of the taxon Tetraconata that is strongly supported in molecular analyses (e.g., [7–9]). In similar fashion, correspondences between the early neurogenic processes in the non-pycnogonid chelicerates (represented by horseshoe crabs and spiders) and myriapods have been briefly discussed as supporting a sister group relationship of both lineages [65–67] but are now more generally considered as the plesiomorphic condition of Arthropoda (e.g., [19, 20]).

When putting the focus on later phases of neurogenesis, VO-like structures as centers of neural cell production have not been reported in any of the recently investigated chelicerate taxa other than Pycnogonida [19, 68, 69], nor are they known from any tetraconate taxon. Intriguingly, however, VO-like cell formations are invariably formed during advanced neurogenesis of the myriapod taxa [70–74], in some studies having even been designated as “ventral organs” [71, 72, 74]. As is the case in pycnogonids, these myriapod VOs develop via an invagination of the central area of the neuroectoderm of each hemisegment. Moreover, in accordance with the here deduced pycnogonid ground pattern, myriapod VOs are subsequently incorporated into the ventral soma cortex of the ganglia, where they have been reported to produce additional ganglion cells [70–73]. In light of increasingly better support for myriapods as sister group of Tetraconata within the taxon Mandibulata (e.g., [7, 27, 75]), we therefore propose internal VOs as centers of late neurogenesis to be a symplesiomorphic feature of gangliogenesis in pycnogonids and myriapods. In other words, we suggest such internalized VOs to be part of the ground pattern of Arthropoda.

Also in the likely arthropod sister group Onychophora, conspicuous segmental thickenings of the ventral ectoderm are formed during advanced development. As for Myriapoda, histological studies have termed these structures “ventral organs” (e.g., [76–78]) and in part suggested their involvement in late embryonic neurogenesis (e.g., [78]). However, several recent investigations have refuted this role (e.g., [79, 18]), as the onychophoran VOs neither invaginate, nor retain a connection to the underlying CNS. Instead, they gradually atrophy to tiny sclerotized epidermal structures that act as muscle attachment sites for limb muscles in the adult animals [79, 80]. Owing to these differences, homologization of these onychophoran structures with the pycnogonid and myriapod VOs seems not tenable. A notable exception to this may be found in the protocerebral neuroectoderm of onychophorans. Here, the central area invaginates during development to form the hypocerebral organ – an external cell cluster that is attached to the ventral side of brain. To this day, the function of the hypocerebral organ remains largely unresolved, and apart from a (neuro)secretory nature (e.g., [37, 81, 82]), a role in late and perhaps even adult neurogenic processes has been considered [22]. Clearly, further studies on this organ are needed for a better understanding of its constituting cell types and their function.

Similarly, it will be the task of future studies to further dissect the cellular processes and their underlying genetic networks in the internal VOs of pycnogonids and myriapods in more detail. For instance, the presence of specialized NSC-like precursors comparable to those in pycnogonids needs to be critically reinvestigated in myriapod taxa, as the currently available data are in this respect still contradictory and inconclusive (e.g., [65, 74, 83]). Beyond that, the potential persistence of internal VOs in the ganglia of adult pycnogonids and myriapods – as shown for the external VOs of *Meridionale* sp. [22] – and the related issue of life-long neurogenic processes in these two arthropod groups remain to be clarified.

## Conclusions

Our comparative study on six different pycnogonid species provides new insights into the evolution of neurodevelopmental processes and adult neuroanatomy in one of the oldest extant arthropod lineages. By evaluating our findings in light of current hypotheses on pycnogonid phylogeny, we reveal a bipartite SEG as the plesiomorphic condition of sea spiders and find evidence for the convergent fusion of walking leg neuromere 1 to this SEG in three different pycnogonid lineages. These fusion events are clearly independent of the apomorphic postoral CNS condensation in the prosoma of euchelicerates (e.g., [12, 84]), as well as of the different SEG formation/expansion events discussed for mandibulate taxa (e.g., [85]). Further, we reconstruct the presence of internal VOs instead of external VOs as plesiomorphic character state of sea spider gangliogenesis. Although chelicerate taxa other than Pycnogonida lack comparable VOs, they are a characteristic feature of myriapod gangliogenesis and, accordingly, we propose internal VOs with neurogenic function to be part of the ground pattern of Arthropoda. Finally, our study demonstrates the importance of an expanded taxon sampling in neurodevelopmental and neuroanatomical investigations, even when dealing with arthropod lineages that show such a conserved basic body organization as Pycnogonida. This enables the documentation of ingroup variation and more reliable differentiation of plesiomorphic from apomorphic character states prior to outgroup comparison.

## Additional files

Additional file 1: Movie of walking leg ganglia 3 and 4 in a young last postlarval instar of *Ph. femoratum*. Labeling of acetylated tubulin (white) and EdU (4 h exposure, red) with nuclear counterstain (blue). Different combinations of the three signals are shown during the movie, in order to show specific aspects in more detail. The movie starts in ventral view, anterior is to the top. The object turns 90° around the a-p axis towards the left to demonstrate that the VOs containing the proliferating cells are embedded in the ventral soma cortex (for better view, one body half is clipped away after the turn). Note the single EdU+ nucleus deep in the cortex of walking leg ganglion 3. After a 90° rotation back into ventral view, a clipping plane is briefly added to show only the ventralmost portion of the soma cortices, which house the strongly tubulin-labeled VOs with their central cavities and the proliferating cells. Note also the presence of EdU+ cells in the posterior ganglion anlagen, which will begin to merge with walking leg ganglion 4 during subsequent development towards the first juvenile instar. (MP4 17,615 kb) (MP4 17615 kb)

Additional file 2: Movie of walking leg ganglion 3 in a juvenile of *Meridionale* sp. Labeling of acetylated tubulin (white), BrdU (green) and EdU (red) (4 h BrdU exposure, 92 h sea water, 4 h EdU exposure, 20 h sea water) with nuclear counterstain (blue). Different combinations of the four signals are shown during the movie to highlight specific aspects in more detail. The movie starts in ventral view, anterior is to the top. The object turns almost 90° around the a-p axis towards the left to demonstrate that the VOs containing the majority of the proliferating cells (as indicated by the BrdU+ and EdU+ nuclei) are located externally on the ventral surface of the ganglion. Only BrdU+ cells are found along the migratory streams that extend into the soma cortex. EdU+ nuclei are located exclusively in the external VOs, showing BrdU co-labeling. Note also the very prominent and curved ventral longitudinal tract that is visible whenever the tubulin signal is shown, e.g., during the rotation back into ventral view at the end of the movie. (MP4 16,957 kb) (MP4 16957 kb)

Additional file 3: Movie of walking leg ganglia 2–4 in the last postlarval instar of *T. orbiculare*. Labeling of acetylated tubulin (white), BrdU (green) and EdU (red) (4 h BrdU exposure, 64 h sea water, 4 h EdU exposure) with nuclear counterstain (blue). Different combinations of the four signals are shown during the movie to highlight specific aspects in more detail. The movie starts in ventral view, anterior is to the top. The object turns 90° around the a-p axis towards the right to demonstrate that the VOs containing the proliferating cells (as indicated by the EdU+ nuclei) are embedded in the ventral soma cortex (for better view, one body half is clipped away after the turn). Note the presence of a higher number of large EdU+ nuclei in the more posterior ganglia and the occurrence of distinctly smaller nuclei in between and around the proliferating VO cells. After the rotation, the BrdU signal is added to demonstrate the predominant double labeling with the EdU+ cells (yellow-orange signal due to overlay of green and red) as well as the wider distribution of BrdU-only cells in the soma cortices. Note the sickle-like arrangement of the BrdU+ cells as they extend and migrate around the growing neuropil in the more anterior walking leg ganglia. Note also the curved ventral longitudinal tract that is at this stage well-differentiated in walking leg ganglion 2, being visible once the tubulin signal is added in lateral view and during the final rotation back into ventral view. (MP4 19,277 kb) (MP4 19277 kb)

Additional file 4: Movie of walking leg ganglia 2 and 3 in a premolting last postlarval instar of *Ph. femoratum.* Labeling of acetylated tubulin (white), BrdU (green) and EdU (red) (6 h BrdU exposure, 12 h sea water, 3 h EdU exposure) with nuclear counterstain (blue). Different combinations of the four signals are shown during the movie, in order to highlight specific aspects more clearly. The movie starts in ventral view, anterior is to the top. Note the more intense nuclear staining of many smaller VO cells. The object turns 90° around the a-p axis towards the right to demonstrate that the VOs containing the proliferating cells (as indicated by the BrdU+ and EdU+ nuclei) are embedded in the ventral soma cortex (for better view, one body half is clipped away after the turn). Note a single dorsal BrdU+ cell that lies close to the segmental nerve root in walking leg ganglion 2. Note also the curved ventral longitudinal tract, which is visible dorsal to the VOs once the tubulin signal is added in lateral view and during the final rotation back into ventral view. The final ventral aspect focuses on walking leg ganglion 3, a clipping plane having been added to remove structures that lie dorsal to the VOs. Switching between the BrdU and EdU channels demonstrates the mixed pattern of BrdU+/EdU+, BrdU+/EdU− and BrdU−/EdU+ nuclei. (MP4 20,632 kb) (MP4 20632 kb)

## Abbreviations

AF: autofluorescence
a-p: anterior-posterior
br: brain
BrdU: 5-bromo-2′-deoxyuridine
ch: cheliphore
CNS: central nervous system
cut: cuticle
EdU: 5-ethynyl-2′-deoxyuridine
ep: epidermis
eso: esophagus
GN: glomerulus-like neuropil
lm: longitudinal muscles
mg: midgut
ot: ocular tubercle
ovn: ovigeral neuromere
pan: palpal neuromere
PH3: phosphorylated histone H3
pha: pharynx
pr: proboscis
SEG/seg: subesophageal ganglion
vlt: ventral longitudinal tract
VNC: ventral nerve cord
VO: ventral organ
wl: walking leg
wlg: walking leg ganglion
wln: walking leg neuromere

## References

[1] Giribet G, Edgecombe GD, Minelli A, Boxshall G, Fusco G (2013). The Arthropoda: a phylogenetic framework. Arthropod biology and evolution molecules, development, morphology.

[2] Edgecombe GD, Legg DA (2014). Frontiers in Palaeontology. Origins and early evolution of arthropods. Palaeontology.

[3] Dohle W (2001). Are the insects terrestrial crustaceans? A discussion of some new facts and arguments and the proposal of the proper name ‘Tetraconata’ for the monophyletic unit Crustacea + Hexapoda. Ann Soc Entomol Fr.

[4] Richter S (2002). The Tetraconata concept: hexapod-crustacean relationships and the phylogeny of Crustacea. Org Divers Evol.

[5] Müller CHG, Rosenberg J, Richter S, Meyer-Rochow VB (2003). The compound eye of *Scutigera coleoptrata* (Linnaeus, 1758) (Chilopoda: Notostigmophora): an ultrastructural reinvestigation that adds support to the Mandibulata concept. Zoomorphology.

[6] Ungerer P, Scholtz G (2008). Filling the gap between identified neuroblasts and neurons in crustaceans adds new support for Tetraconata. Proc R Soc B.

[7] Regier JC, Shultz JW, Zwick A, Hussey A, Ball B, Wetzer R, Martin JW, Cunningham CW (2010). Arthropod relationships revealed by phylogenomic analysis of nuclear protein-coding sequences. Nature.

[8] von Reumont BM, Jenner RA, Wills MA, Dell’Ampio E, Pass G, Ebersberger I, Meyer B, Koenemann S, Iliffe TM, Stamatakis A (2012). Pancrustacean phylogeny in the light of new phylogenomic data: support for Remipedia as the possible sister group of Hexapoda. Mol Biol Evol.

[9] Schwentner M, Combosch D, Nelson J, Giribet G (2017). A phylogenomic solution to the origin of insects by resolving crustacean-hexapod relationships. Curr Biol.

[10] Harzsch S (2006). Neurophylogeny: architecture of the nervous system and a fresh view on arthropod phylogeny. Integr Comp Biol.

[11] Strausfeld NJ, Andrew DR (2011). A new view of insect-crustacean relationships I. Inferences from neural cladistics and comparative neuroanatomy. Arthropod Struct Dev.

[12] Strausfeld NJ (2012). Arthropod brains. Evolution, functional elegance, and historical significance. Cambridge (Massachusetts).

[13] Loesel R, Wolf H, Kenning M, Harzsch S, Sombke A, Minelli A, Boxshall G, Fusco G (2013). Architectural principles and evolution of the arthropod central nervous system. Arthropod biology and evolution molecules, development, morphology.

[14] Strausfeld NJ, Ma X, Edgecombe GD (2016). Fossils and the evolution of the arthropod brain. Curr Biol.

[15] Sombke A, Stemme T (2017). Serotonergic neurons in the ventral nerve cord of Chilopoda - a mandibulate pattern of individually identifiable neurons. Zoological Lett.

[16] Martin C, Gross V, Hering L, Tepper B, Jahn H, Oliveira IS, Stevenson PA, Mayer G (2017). The nervous and visual systems of onychophorans and tardigrades: learning about arthropod evolution from their closest relatives. J Comp Physiol A.

[17] Wolff G, Thoen H, Marshall J, Sayre M, Strausfeld NJ (2017). An insect-like mushroom body in a crustacean brain. elife.

[18] Whitington PM, Mayer G (2011). The origins of the arthropod nervous system: insights from the Onychophora. Arthropod Struct Dev.

[19] Brenneis G, Stollewerk A, Scholtz G (2013). Embryonic neurogenesis in *Pseudopallene* sp. (Arthropoda, Pycnogonida) includes two subsequent phases with similarities to different arthropod groups. EvoDevo.

[20] Stollewerk A (2016). A flexible genetic toolkit for arthropod neurogenesis. Philos Trans R Soc Lond.

[21] Staples D (2014). A revision of the callipallenid genus *Pseudopallene* Wilson, 1878 (Pycnogonida, Callipallenidae). Zootaxa.

[22] Brenneis G, Scholtz G (2014). The ‘ventral organs’ of Pycnogonida (Arthropoda) are neurogenic niches of late embryonic and post-embryonic nervous system development. PLoS One.

[23] Rudkin DM, Cuggy MB, Young GA, Thompson DP (2013). An Ordovician pycnogonid (sea spider) with serially subdivided ‘head’ region. J Paleontol.

[24] Waloszek D, Dunlop JA (2002). A larval sea spider (Arthropoda: Pycnogonida) from the upper Cambrian “Orsten” of Sweden, and the phylogenetic position of pycnogonids. Palaeontology.

[25] Dunlop JA (2010). Geological history and phylogeny of Chelicerata. Arthropod Struct Dev.

[26] Dunlop JA, Arango CP (2005). Pycnogonid affinities: a review. J Zool Syst Evol Res.

[27] Rehm P, Meusemann K, Borner J, Misof B, Burmester T (2014). Phylogenetic position of Myriapoda revealed by 454 transcriptome sequencing. Mol Phylogenet Evol.

[28] Sharma PP, Kaluziak ST, Pérez-Porro AR, González VL, Hormiga G, Wheeler WC, Giribet G (2014). Phylogenomic interrogation of Arachnida reveals systemic conflicts in phylogenetic signal. Mol Biol Evol.

[29] Schwager EE, Schönauer A, Leite DJ, Sharma PP, McGregor AP, Wanninger A (2015). Chelicerata. Evolutionary developmental biology of invertebrates volume 3 Ecdysozoa I: non-Tetraconata.

[30] Helfer H, Schlottke E (1935). Pantopoda. Bronns Klassen und Ordnungen des Tierreichs, Bd. 5, Abt. IV, Buch 2.

[31] Henry LM (1953). The nervous system of the Pycnogonida. Microentomology.

[32] Brenneis G. Pycnogonida (Pantopoda). In: Schmidt-Rhaesa A, Harzsch S, Purschke G, editors. Structure and evolution of invertebrate nervous systems. Oxford: Oxford University Press; 2016. p. 419–27.

[33] Morgan TH (1891). A contribution to the embryology and phylogeny of the pycnogonids.

[34] Meisenheimer J (1902). Beiträge zur Entwicklungsgeschichte der Pantopoden. I. Die Entwicklung von *Ammothea echinata* Hodge bis zur Ausbildung der Larvenform. Z Wiss Zool.

[35] Dogiel V (1911). Studien über die Entwicklungsgeschichte der Pantopoden. Nervensystem und Drüsen der Pantopodenlarven. Z Wiss Zool.

[36] Dogiel V (1913). Embryologische Studien an Pantopoden. Z Wiss Zool.

[37] Sanchez S (1958). Cellules neurosécrétrices et organes infracérébraux de *Peripatopsis moseleyi* Wood (Onychophores) et neurosécrétion chez *Nymphon gracile* Leach (Pycnogonides). Arch Zool Exp Gén Notes Revue.

[38] Sanchez S (1959). Le développement des Pycnogonides et leurs affinités avec les Arachnides. Arch Zool Exp Gén.

[39] Winter G (1980). Beiträge zur Morphologie und Embryologie des vorderen Körperabschnitts (Cephalosoma) der Pantopoda Gerstaecker, 1863. I. Entstehung und Struktur des Zentralnervensystems. Z Zool Syst Evol.

[40] Arango CP, Wheeler WC (2007). Phylogeny of the sea spiders (Arthropoda, Pycnogonida) based on direct optimization of six loci and morphology. Cladistics.

[41] Arabi J, Cruaud C, Couloux A, Hassanin A (2010). Studying sources of incongruence in arthropod molecular phylogenies: sea spiders (Pycnogonida) as a case study. C R Biol.

[42] Brenneis G, Bogomolova EV, Arango CP, Krapp F (2017). From egg to “no-body”: an overview and revision of developmental pathways in the ancient arthropod lineage Pycnogonida. Front Zool.

[43] Bamber RN, El Nagar A, Arango CP. Pycnobase: World Pycnogonida Database. Available online at http://www.marinespecies.org/pycnobase. Accessed 13 March 2018.

[44] Lovely EC (2005). The life history of *Phoxichilidium tubulariae* (Pycnogonida: Phoxichilidiidae). Northeast Nat.

[45] Arango CP, Brenneis G (2013). New species of Australian *Pseudopallene* (Pycnogonida: Callipallenidae) based on live colouration, morphology and DNA. Zootaxa.

[46] Behrens W (1984). Larvenentwicklung und metamorphose von *Pycnogonum litorale* (Chelicerata, Pantopoda). Zoomorphology.

[47] Tomaschko KH, Wilhelm E, Bückmann D (1997). Growth and reproduction of *Pycnogonum litorale* (Pycnogonida) under laboratory conditions. Mar Biol.

[48] Vilpoux K, Waloszek D (2003). Larval development and morphogenesis of the sea spider *Pycnogonum litorale* (Ström, 1762) and the tagmosis of the body of Pantopoda. Arthropod Struct Dev.

[49] Ungerer P, Scholtz G (2009). Cleavage and gastrulation in *Pycnogonum litorale* (Arthropoda, Pycnogonida): morphological support for the Ecdysozoa?. Zoomorphology.

[50] Brenneis G, Scholtz G (2015). Serotonin-immunoreactivity in the ventral nerve cord of Pycnogonida – support for individually identifiable neurons as ancestral feature of the arthropod nervous system. BMC Evol Biol.

[51] Brenneis G, Arango CP, Scholtz G (2011). Morphogenesis of *Pseudopallene* sp. (Pycnogonida, Callipallenidae) II: postembryonic development. Dev Genes Evol.

[52] Loman JCC (1917). Beiträge zur Anatomie und Biologie der Pantopoden. Tijdschr Ned Dierkundige Vereeniging Ser 2.

[53] Sabroux R, Corbari L, Krapp F, Bonillo C, Le Prieur S, Hassanin A (2017). Biodiversity and phylogeny of Ammotheidae (Arthropoda: Pycnogonida). Eur J Taxon.

[54] Hoek PPC (1881). Report on the Pycnogonida, dredged by H.M.S. challenger during the years 1873-76. Challenger report. Zoology.

[55] Dohrn A (1881). Die Pantopoden des Golfes von Neapel und der angrenzenden Meeres-Abschnitte.

[56] Nakamura K, Kano Y, Suzuki N, Namatame T, Kosaku A (2007). 18S rRNA phylogeny of sea spiders with emphasis on the position of Rhynchothoracidae. Mar Biol.

[57] Alexeeva N, Bogomolova EV, Tamberg Y, Shunatova N (2017). Oligomeric larvae of the pycnogonids revisited. J Morphol.

[58] Bogomolova EV, Malakhov VV (2003). Larvae of sea spiders (Arthropoda, Pycnogonida) from the White Sea. Entomol Rev.

[59] Gillespie JM, Bain BA (2006). Postembryonic development of *Tanystylum bealensis* (Pycnogonida, Ammotheidae) from Barkley sound, British Columbia, Canada. J Morphol.

[60] Maxmen A (2006). Pycnogonid development and the evolution of the arthropod body plan.

[61] Burris ZP (2011). Larval morphologies and potential developmental modes of eight sea spider species (Arthropoda: Pycnogonida) from the southern Oregon coast. J Mar Biol Assoc U K.

[62] Cano Sánchez E, López-González PJ (2013). New data concerning postembryonic development in Antarctic *Ammothea* species (Pycnogonida: Ammotheidae). Polar Biol.

[63] Hübner J, Wagner P, Lehmann T, Melzer RR (2017). Testing species delimitation with larval morphology: scanning electron microscopy analysis of protonymphon larvae of two closely related sea spiders, *Pallenopsis patagonica* (Hoek) and *Pallenopsis yepayekae* Weis. Invertebr Syst.

[64] Ungerer P, Eriksson BJ, Stollewerk A (2011). Neurogenesis in the water flea *Daphnia magna* (Crustacea, Branchiopoda) suggests different mechanisms of neuroblast formation in insects and crustaceans. Dev Biol.

[65] Dove H, Stollewerk A (2003). Comparative analysis of neurogenesis in the myriapod *Glomeris marginata* (Diplopoda) suggests more similarities to chelicerates than to insects. Development.

[66] Kadner D, Stollewerk A (2004). Neurogenesis in the chilopod *Lithobius forficatus* suggests more similarities to chelicerates than to insects. Dev Genes Evol.

[67] Mayer G, Whitington PM (2009). Velvet worm development links myriapods with chelicerates. Proc R Soc B.

[68] Stollewerk A, Weller M, Tautz D (2001). Neurogenesis in the spider *Cupiennius salei*. Development.

[69] Mittmann B (2002). Early neurogenesis in the horseshoe crab *Limulus polyphemus* and its implication for arthropod relationships. Biol Bull.

[70] Heymons R (1901). Die Entwicklungsgeschichte der Scolopender. Zoologica.

[71] Tiegs OW (1940). The embryology and affinities of the Symphyla, based on a study of *Hanseniella agilis*. Q J Microsc Sci.

[72] Tiegs OW (1947). The development and affinities of the Pauropoda, based on a study of *Pauropus silvaticus*. Part I. Q J Microsc Sci.

[73] Dohle W (1964). Die Embryonalentwicklung von *Glomeris marginata* (Villers) im Vergleich zur Entwicklung anderer Diplopoden. Zool Jahrb Anat.

[74] Knoll HJ (1974). Untersuchungen zur Entwicklungsgeschichte von *Scutigera coleoptrata* L. (Chilopoda). Zool Jahrb Anat.

[75] Lozano-Fernandez J, Carton R, Tanner AR, Puttnick MN, Blaxter M, Vinther J, Olesen J, Giribet G, Edgecombe GD, Pisani D (2016). A molecular palaeobiological exploration of arthropod terrestrialization. Philos Trans R Soc B.

[76] Kennel J (1886). Entwicklungsgeschichte von *Peripatus edwardsii* Blanch. und *Peripatus torquatus n.sp*.

[77] Sedgwick A. A monograph of the development of *Peripatus capensis*. In: Studies from the morphological Laboratory in the University of Cambridge. Cambridge: Cambridge University Press; 1888. p. 1–146.

[78] Pflugfelder O (1948). Entwicklung von *Paraperipatus amboinensis* n. spec. Zool Jahrb Anat.

[79] Mayer G, Whitington PM (2009). Neural development in Onychophora (velvet worms) suggests a step-wise evolution of segmentation in the nervous system of Panarthropoda. Dev Biol.

[80] de Sena Oliveira I, Tait NN, Strübing I, Mayer G (2013). The role of ventral and preventral organs as attachment sites for segmental limb muscles in Onychophora. Front Zool.

[81] Eriksson BJ, Tait NN, Norman JM, Budd GE (2005). An ultrastructural investigation of the hypocerebral organ of the adult *Euperipatoides kanangrensis* (Onychophora, Peripatopsidae). Arthropod Struct Dev.

[82] Eriksson BJ, Samadi L, Schmid A (2013). The expression pattern of the genes *engrailed*, *pax6*, *otd* and *six3* with special respect to head and eye development in *Euperipatoides kanangrensis* Reid 1996 (Onychophora: Peripatopsidae). Dev Genes Evol.

[83] Whitington PM, Meier T, King P (1991). Segmentation, neurogenesis and formation of early axonal pathways in the centipede, *Ethmostigmus rubripes* (Brandt). Rouxs Arch Dev Biol.

[84] Scholtz G. Perspective---heads and brains in arthropods: 40 years after the ‘Endless dispute’. In: Schmidt-Rhaesa A, Harzsch S, Purschke G, editors. Structure and evolution of invertebrate nervous systems. Oxford: Oxford University Press; 2016.

[85] Stegner MEJ, Brenneis G, Richter S (2014). The ventral nerve cord in Cephalocarida (Crustacea): new insights into the ground pattern of Tetraconata. J Morphol.

